# Patients’ Needs Regarding Work-Focused Healthcare: A Qualitative Evidence Synthesis

**DOI:** 10.1007/s10926-024-10225-8

**Published:** 2024-07-25

**Authors:** Marije E. Hagendijk, Nina Zipfel, Marijke Melles, Philip J. van der Wees, Carel T. J. Hulshof, Elmi Zwaan, Sylvia J. van der Burg-Vermeulen, Jan L. Hoving

**Affiliations:** 1 Department of Public and Occupational Health, Coronel Institute of Occupational Health, Amsterdam Public Health Research Institute, Amsterdam UMC, University of Amsterdam, Meibergdreef 9, 1105 AZ Amsterdam, The Netherlands; 2 Faculty of Industrial Design Engineering, Delft University of Technology, Delft, The Netherlands; 3 Scientific Institute for Quality of Healthcare (IQ Healthcare), Radboud Institute for Health Sciences, Radboud University Nijmegen Medical Centre, Nijmegen, The Netherlands; 4 Dutch Research Center for Insurance Medicine, Amsterdam, The Netherlands

**Keywords:** Occupational health, Return to work, Sick-leave, Chronic disease, Qualitative research, Systematic review, Delivery of healthcare

## Abstract

**Purpose:**

To identify, appraise, and synthesize qualitative research evidence exploring patients’ needs regarding work-focused healthcare.

**Methods:**

A systematic review was conducted in accordance with the PRISMA statement guidelines to identify studies reporting patients’ needs regarding work-focused healthcare. Four databases (MEDLINE, Embase, PsychInfo and Web of Science) were systematically searched from January 2000 until May 2023 and screened in duplicate by pairs of two reviewers. Inclusion criteria were qualitative data collection method, and patients’ perspectives regarding healthcare focusing on work when experiencing work-related problems due to chronic medical conditions. Data extraction and synthesis was executed by means of an inductive thematic analysis approach. The quality of the included studies was assessed using the CASP Qualitative Study checklist. Confidence in the review findings was assessed through the Confidence in the Evidence from Reviews of Qualitative research (CERQual) approach.

**Results:**

Out of 23,677 records, 97 qualitative studies were included. Needs regarding four main themes were identified: (1) Substantive guidance, which comprises the specific content of work-focused healthcare; (2) Clear and continuous process, which comprises clarification and optimization of the work-focused healthcare process; (3) Supportive attitude and behavior, which comprises a positive and supportive attitude and behavior from professionals towards the patients; and (4) Tailored approach, which comprises the delivery of tailored care to the individuals’ needs. 17 subthemes were identified.

**Conclusion:**

The broader insight in patients’ needs in work-focused healthcare can help (occupational) healthcare professionals adopt a more patient-centred approach in practice.

**Supplementary Information:**

The online version contains supplementary material available at 10.1007/s10926-024-10225-8.

## Introduction

Recent years have seen an increase in the number of people with chronic medical conditions and the average age of the working population worldwide [1], which can be explained by rising retirement ages [1–3] and declining mortality rates in the working population [4]. Chronic medical conditions can negatively affect the individual’s work ability in both the short- and long-term [5, 6]. Work disability, resulting in sick-leave, unemployment or disability benefit, often leads to a decline in all facets of overall health-related quality of life, with social and emotional functioning being particularly affected [7]. For individuals facing work disability the ability to stay at work (SAW) or successfully return to work (RTW) is of paramount importance.

However, individuals living with medical conditions often encounter numerous barriers to SAW or RTW that they cannot overcome alone [8, 9]. In such cases, interventions like vocational rehabilitation, as well as guidance and support from (occupational) healthcare professionals and authorities have been identified as facilitators to overcome work participation problems [10]. The support and guidance provided by (occupational) healthcare professionals, and relevant authorities, focusing on work-related concerns and obstacles, is referred to as work-focused healthcare [11]. Nonetheless, individuals receiving work-focused healthcare, hereafter called patients, frequently express dissatisfaction with the delivery of such services, citing unwanted support or inadequate provision of crucial information [6, 12].

In accordance with the value-based healthcare concept, embracing a patient-centred approach within the healthcare system, enhances the value for the patient by better addressing their preferences and needs [13, 14]. Thereby, adopting better patient-centred work-focused healthcare delivery is suggested to also enhance patient satisfaction in work-focused healthcare [15, 16]. However, a deep understanding of patients’ needs within work-focused healthcare is needed to effectively implement a patient-centred approach within work-focused healthcare [16]. In addition, not only practitioners could benefit from recognizing these patients’ needs for work focused healthcare, also researchers could identify research gaps in areas where these needs are not met.

Although there is an increasing amount of qualitative literature exploring patients’ perspectives on work-focused healthcare, a comprehensive overview is currently absent. Therefore, the objective of this systematic review was to identify, appraise, and synthesize qualitative research evidence on patients’ needs regarding integrated work-focused healthcare when experiencing problems with work participation due to a medical condition.

## Methods

The protocol for this qualitative evidence synthesis has been published on the PROSPERO platform (ID: CRD42021232699). The thematic analysis approach of Thomas and Harden (2008) was used for the data extraction and synthesis. To report this review the Enhancing transparency in reporting the synthesis of qualitative research (ENTREQ) checklist was used [17].

### Data Sources and Searches

The search strategy was developed by an experienced clinical librarian from the Amsterdam UMC/AMC Medical Library. The strategy was formulated through the utilization of a test sample of relevant studies and initial search terms provided by the reviewers. The initial search strategy was further enriched and tested through subsequent sessions between the librarian and three reviewers (MH, SB, JH). The librarian developed and validated the final search strategy in accordance with the nine criteria of the Canadian Agency for Drugs and Technologies in Healthcare Peer Review Checklist for Search Strategies. The search strategy developed included terms related to challenges concerning work participation and work functioning, qualitative research, and separate terms for *patient* and *perspective* linked with an adjacent operator. This search strategy was tailored to multiple databases: MEDLINE, Embase, PsychInfo, and two conference proceedings Citations of the Web of Science (Citation Index Science & Citation Index Social Sciences and Humanities), searched from January 2000 until the 27th of May 2023. This time frame was selected because we hypothesized that there would be a scarcity of qualitative records on the subject before 2000 and we wanted to reflect more the current state of practice and healthcare. The full search strategy is presented in the online Supplementary Appendix Table 1. Relevant conference abstracts extracted from the conference proceedings were searched for their full text.

### Study Selection

The study inclusion criteria comprised the following: (i) qualitative study design using individual interviews and/or focus groups for data collection; (ii) participants of working age living with a chronic medical condition; (iii) exploration of work-related challenges arising from a (chronic) medical condition, such as work disability, sickness absence, unemployment, issues during SAW or RTW; (iv) inclusion of at least one outcome regarding patients’ experiences and/or needs concerning work-focused guidance from (occupational) healthcare professionals; and (v) articles written in English. There were no geographic restrictions. Mixed-method studies were included if qualitative data could be extracted separately. Similarly, primary studies considering multiple stakeholder perspectives were also considered.

Prior to the screening process, duplicate articles and those published before 2000 were excluded. The screening process involved three main steps [18]. First, a single author (MH) screened the articles for relevance based on the title. Second, pairs of authors (MH or NZ and SB, JH, MM, PW, EZ or CH) independently assessed the title and abstract of the remaining articles using the inclusion criteria. Prior to this assessment, a pilot screening was performed independently by authors for a random selection of fifty articles. Third, for the studies identified after title and abstract screening, a duplicate full-text screening was performed by the same author pairs. Conflicts during the second and third steps were resolved through pair discussion until consensus was reached. Any remaining disagreements were resolved by discussion with a third author (MH or NZ). The Rayyan online systematic review screening tool was used as the technical platform throughout the screening process [19].

### Data Extraction and Quality Assessment

For data extraction the thematic synthesis method of Thomas and Harden’s was adopted, starting with line by line coding [20]. During the line by line coding, the first author (MH) assigned individual codes to indicated needs and experiences reflecting on specific needs. A single author (MH or EZ) extracted the study characteristics, such as author, publication year, country, study aim, and participant details, using Microsoft Access.

The quality of each included article was assessed by two authors independently (MH or NZ and SB, JH, MM, PW, EZ or CH) using the Critical Appraisal Skills Programme (CASP) qualitative checklist [21]. The CASP checklist includes 10 items to appraise the quality of qualitative research [21]. Articles meeting eight or more criteria were rated as high quality, those meeting five to seven criteria as medium quality, and those meeting four or less as low [22]. Studies were not excluded based on their assessed quality. Differences in assessment were discussed within the pairs until consensus was reached. Authors of the current study who were associated with any included article were not involved in assessing its quality to prevent conflict of interest.

### Data Synthesis and Analysis

As described by Thomas and Harden [20], after the data extraction through line by line coding, the data synthesis consisted of two main stages: identifying descriptive themes and generating analytical themes. The first author (MH) derived the descriptive themes directly from the primary studies, while analytical themes required interpretation and explanatory constructs [20]. Themes and subcategories were developed inductively. Two co-authors (NZ and EZ) randomly checked the coding system during the line by line and descriptive coding. The final coding system, developed during analytical coding, was discussed and confirmed during multiple meetings with all authors. The MAXQDA plus 2020 software was used to assist the data extraction and synthesis.

The confidence of each finding was assessed with the Confidence in the Evidence from Reviews of Qualitative research (CERQual) approach [23], using the GRADE-CERQual Interactive Summary of Qualitative Findings (iSoQ) computer program [24]. This approach is becoming the standard in assessing the confidence in findings of a systematic review of qualitative research [25]. CERQual assesses the confidence in the evidence based on (i) methodological limitations [26], (ii) coherence [27], (iii) data adequacy [28], and (iv) relevance [29]. After assessing the degree of concern of each of the four components, the overall confidence of each review finding was judged to be high, moderate, low or very low. High confidence suggests that it is highly likely that the review finding is a reasonable representation of the phenomenon of interest, while very low confidence indicates that it is not clear whether the review finding is a reasonable representation of the phenomenon of interest [25]. The assessment was performed by one author (MH), checked by another author (NZ or JH), and finalized after consensus with four authors (MH, NZ, JH, SB).

## Results

### Studies Included

**Fig. 1:**
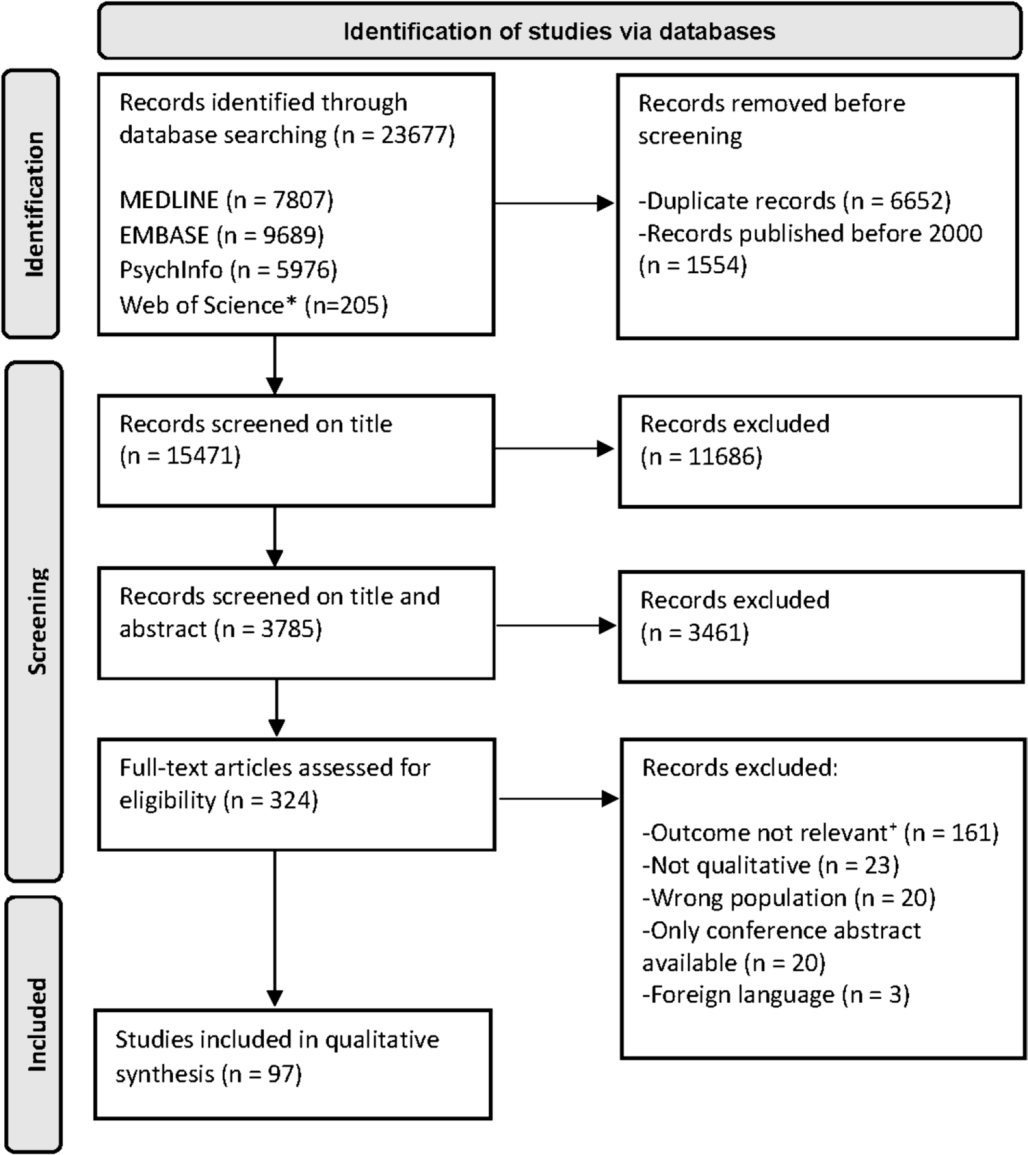
PRISMA 2020 flow diagram. *Two conference proceedings Citations of the Web of Science (Citation Index Science & Citation Index Social Sciences and Humanities), ^+^No needs or experiences which reflect on a certain need regarding the topic of this paper

A total of 23,677 studies were identified, of which 97 studies met our inclusion criteria. The search and selection process is presented in Fig. 1. The 97 qualitative studies, each representing between *n* = 5 and *n* = 73 participants, included in total *n* = 1817 participants experiencing problems with work participation due to a chronic medical condition. The included studies had a wide range of chronic medical conditions, including cancer (*n* = 24), brain injury (*n* = 11), mental illness (*n* = 10), cardiovascular problems (*n* = 8), back pain (*n* = 7), knee replacement (*n* = 4), arthritis (*n* = 4), other (*n* = 10), and studies including a specific patient population with a wide range of chronic conditions (*n* = 19). In addition, the work status of the populations in the included studies were: (1) being on (long-term) sick-leave (*n* = 11); (2) coping with problems with work participation while staying at work (*n* = 5); and (3) returned to work after sick-leave (*n* = 22). A combination of these groups was included in *n* = 52 of the studies, and for the participants from *n* = 7 included studies the current work status was unknown. An overview of all study characteristics of each study is shown in the online Supplementary Appendix Table 2. The results of the CASP qualitative checklist for each study is presented in the online Supplementary Appendix Table 3. Of the included studies, *n* = 62 (63.9%) studies were rated high-quality studies (8-10), *n* = 33 (34.0%) studies medium quality (5-7), and *n* = 2 (2.1%) studies low quality (0–4) [22].

### Identified Needs

**Fig. 2:**
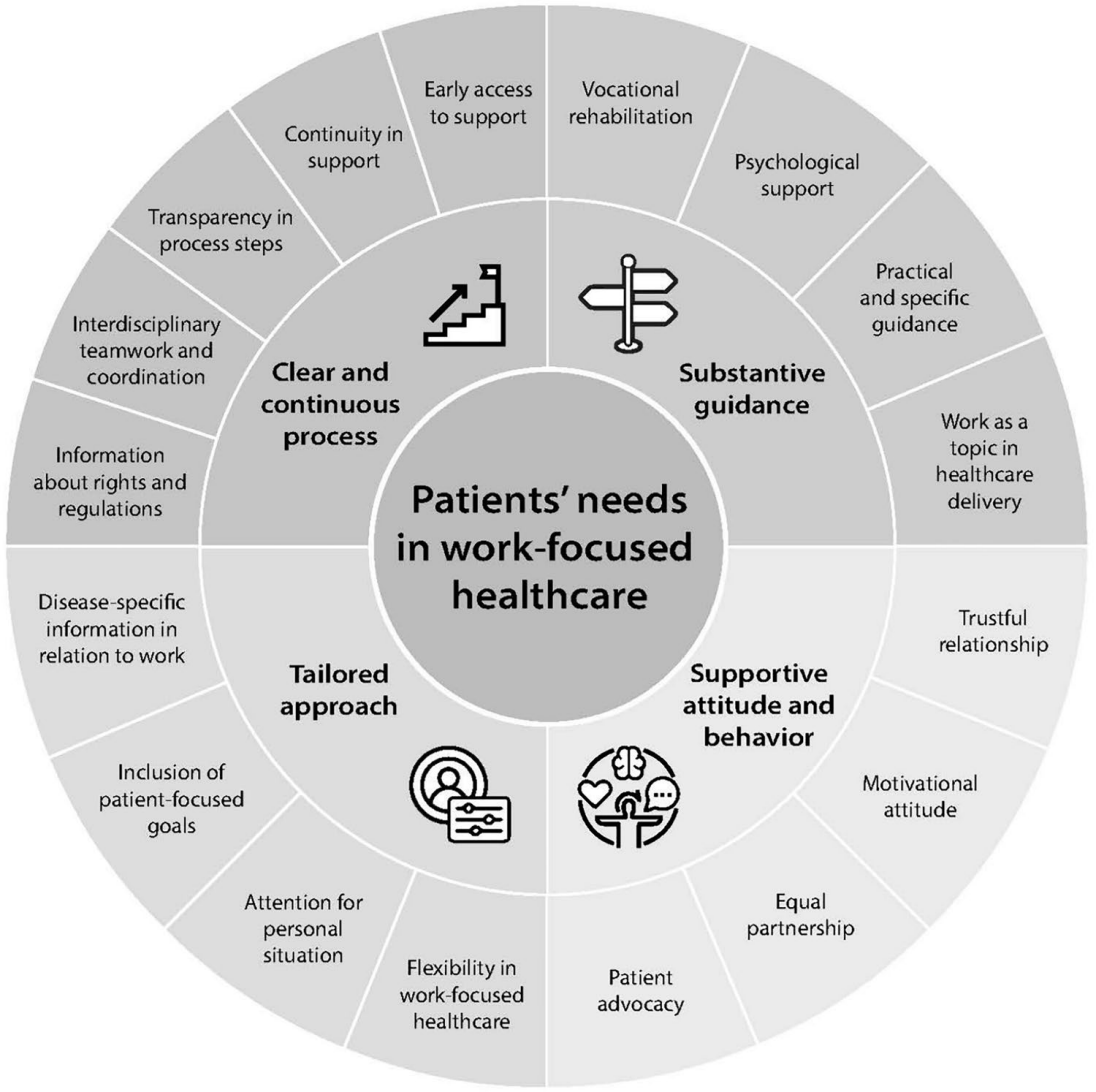
Concept map of the identified patients’ needs in work-focused healthcare

A broad variety of needs regarding work-focused healthcare as addressed by participating patients were identified, displayed in an overview of 17 subthemes. These 17 subthemes were inductively subdivided into four main themes: 1. substantive guidance; 2. clear and continuous process; 3. supportive attitude and behavior; and 4. tailored approach. Hereby, a great variety of healthcare professionals involved in the delivery of work-focused healthcare were mentioned by participating patients. See the online Supplementary Appendix Table 2 for information about the reported professionals per included study. We will discuss below each of the four main themes and their subthemes. An overview of the identified main themes and subthemes, including the brief description for each subtheme, can be found in Table 1. A concept map of the identified themes and subthemes can be found in Fig. 2.

**Table 1:** Overview of the needs regarding work-focused healthcare from the patient’s perspective

Main theme Needs regarding:	Subtheme Identified needs:		Brief description of the need Need for…
1. Substantive guidance	1.1	Work as a topic in healthcare delivery	Work-focused support by all professionals throughout the healthcare delivery process, including the medical specialist and rehabilitation professional, to facilitate staying at work or returning to work
	1.2	Practical and specific guidance	Receive practical tips, e.g. on work modifications, and targeted and phased rehabilitation and return to work plans including realistic goals, in order to help the patient avoid exceeding their limits
	1.3	Psychological support	Psychological assessment and support to help process the impact of the medical condition on impairment in living and working
	1.4	Vocational rehabilitation	Vocational rehabilitation to gain insight into and restore functional abilities and to explore suitable work arrangements
2. Clear and continuous process	2.1	Early access to support	Early presence and access to work-focused healthcare support, by being able to easily reach out and make timely appointments with relevant professionals
	2.2	Continuity in support	Continuous work-focused consultations, including continuous presence of support after full return to work, and the option to fall back on someone when struggling with problems with work participation
	2.3	Transparency in the process steps	Transparency in the multiple process steps, for example by offering a clear overview of the role and responsibility of each professional in the process and clear feedback on how decisions affect the process
	2.4	Interdisciplinary teamwork and coordination	Coherent interaction and constructive collaboration between professionals involved in work-focused healthcare, as well as towards the employer. Involvement of an independent mediator to coordinate the process is suggested
	2.5	Information about rights and regulations	A clear overview of the rights and regulations regarding the work-focused healthcare process and the patient’s obligations, in different formats at multiple time points throughout the process
3. Supportive attitude and behavior	3.1	Trustful relationship	A trustful relationship with the professional, developed by being treated with respect, taken seriously, being trusted, and an emphatic and in-person approach from the professional
	3.2	Motivational attitude	An encouraging, positive, and proactive attitude from professionals, by sharing positive thoughts about the patient’s abilities, to motivate the patient to return to work
	3.3	Equal partnership	An equal partnership, with equal power dynamics, between the professional and patient in making decisions regarding vocational reintegration, by listening and valuing the patient’s choices
	3.4	Patient advocacy	The professional to act in the patient’s interests instead of in the interests of other parties, such as the employer
4. Tailored approach	4.1	Flexibility in work-focused healthcare	Flexibility in the work-focused healthcare provision, and flexibility in the application of the rules in the context of the patient’s needs, in order to receive more tailored support
	4.2	Attention for the personal situation	Attention for the personal situation, including understanding of work capabilities and knowledge of the specific medical situation, on the part of the professional
	4.3	Inclusion of patient-focused goals	Inclusion of patient-focused goals, meeting the patient’s own goals and motivation
	4.4	Disease-specific information in relation to work	Information provision on the expected disease-specific consequences on work, such as expected return to work timelines and impact on work-capacity due to the diagnosis

#### Substantive Guidance

The theme *substantive guidance* comprises the needs regarding the specific content of work-focused healthcare provision. The subthemes represent the identified needs for: work as a topic in healthcare delivery, practical and specific guidance, psychological support, and vocational rehabilitation.

##### Work as a Topic in Healthcare Delivery

Need for work-focused support by all professionals throughout the healthcare delivery process, including the medical specialist and rehabilitation professional, to facilitate staying at work or returning to work (CERQual assessment: high confidence).

Receiving work-focused healthcare support was pointed out as being necessary to SAW or RTW [30–33]. When patients experienced a deficiency in work-focused healthcare support, they reported longer durations of being on sick leave [34–36]. Patients indicated the need for incorporating work as a topic within their clinical treatment [36–58] and throughout their rehabilitation programs [59–63]. The absence of such integration gave patients the feeling of being on their own [49, 64]. Additionally, patients indicated to highly value the opinion of medical specialists and general practitioners regarding their possibilities to RTW [39, 55, 60, 65–67]. In this context, patients indicated to feel responsible for integrating the topic of work into the consultation with the medical specialist [54]. However, patients experienced a feeling of not knowing how to start the conversation about work-related challenges and ask the right question to understand the information given [54, 68, 69]. Therefore, information about how to communicate with professionals on work-related matters was identified as a need [70].

##### Practical and Specific Guidance

Need to receive practical tips, e.g. on work modifications, and targeted and phased rehabilitation and return to work plans including realistic goals, in order to help the patient avoid exceeding their limits (CERQual assessment: high confidence).

A need for explicit and specific advice was identified [49, 50, 69, 71–73], since the absence of advice when discussing work [49, 74] or receiving advice without explicit and specific advice gave patients a feeling of uncertainty [37, 41, 44, 60, 75, 76]. In particular, specific rehabilitation [35, 39, 62, 77–79] and phased RTW plans [34, 35, 37, 39, 72, 75, 77, 80–84] were mentioned, including specific advice about when and how to RTW [48, 68, 72, 85]. In this context, it was mentioned that it is extremely important to set realistic goals with objective measurable outcomes [30]. In addition, practical advice on work modifications [12, 32, 35, 44, 45, 49, 50, 70, 75, 77, 79, 86, 87], assessment at the work-site [30, 75] and advice on finding a balance between social and working life patterns [50, 61, 77, 79] can help patients to avoid exceeding their work ability.

##### Psychological Support

Need for psychological assessment and support to help process the impact of the medical condition on impairment in living and working (CERQual assessment: high confidence).

A need for psychological support was identified [43, 62, 67, 88, 89], since cognitive support was considered particularly helpful in order to feel mentally strong enough to RTW [32, 72, 83, 84]. Psychological support was mentioned as being helpful when providing: support in accepting, and adjusting to, living and working with the chronic medical condition [31, 70, 80, 88–91]; strategies to overcome negative thoughts [30, 42]; and learning how to set boundaries to avoid exceeding physical abilities [70]. In addition to this need for psychological support from a psychologist, the need for psychological assessment by other professionals was identified [32, 59, 72, 89, 92, 93]. Support groups connecting patients with similar experiences and involvement in patient interest organizations were also considered helpful in accepting and adjusting [30, 32, 40, 59]. In addition to psychological support for themselves, patients also indicated the need for psychological support for their families [88].

##### Vocational Rehabilitation

Need for vocational rehabilitation to gain insight into and restore functional abilities and to explore suitable work arrangements (CERQual assessment: moderate confidence).

Vocational rehabilitation, including opportunities to explore varied work tasks and undergo physical assessment, could give patients insight into their altered work capabilities and aid them in finding a suitable job [45, 75, 77, 94, 95]. For patients with physical limitations, such as after total knee arthroplasty, support from physiotherapy was highly appreciated to restore functional capacities in order to facilitate RTW [60]. Unemployed patients indicated a need for information about companies that hire patients who experience problems with work participation [45, 70, 96]. For employed patients such information was also deemed important to facilitate a job switch that aligned with their adjusted work ability [42, 62, 63, 74, 97].

#### Clear and Continuous Process

The theme *clear and continuous process* comprises the needs focusing on clarifying and optimizing the work-focused healthcare process. The subthemes represent the identified needs for: early access to support, continuity in support, transparency in the process steps, interdisciplinary teamwork and coordination, and information about rights and regulations.

##### Early Access to Support

Need for early presence and access to work-focused healthcare support, by being able to easily reach out and make timely appointments with relevant professionals (CERQual assessment: moderate confidence).

Participating patients indicated that work-focused support should be initiated as soon as possible [31, 43, 77, 84, 98, 99], at the latest prior to the start of complex problems [42, 87]. This results in the needs for the early presence of vocational rehabilitation [30, 45, 63, 71, 88, 89] and work-focused coaching directly after discharge [87, 88, 100]. It is thereby highly important to be aware of the available types of support [46, 70, 73], and be able to easily reach out [42, 59, 63, 70, 75, 77, 79, 84, 89, 92, 101] and make a timely appointment with the relevant professional [60, 79], even for self-employed workers [42], to avoid a feeling of isolation [31]. However, professionals from the occupational health services should keep in mind that an invitation for a consultation soon after onset of work participation problems can give the patient a feeling of distrust and lack of understanding for their situation [91].

##### Continuity in Support

Need for continuous work-focused consultations, including continuous presence of support after full return to work, and the option to fall back on someone when struggling with problems with work participation (CERQual assessment: moderate confidence).

A low frequency of guidance, including an early and unforeseen discontinuation of support, led to feelings of confusion, uncertainty and a feeling of being overlooked among patients [8, 40, 41, 59, 71, 89, 94, 102, 103]. Additionally, since patients indicated to wait until their next consultation before deciding on the next step [39], the need for continuous support, including frequent follow-up consultations [104] and automatically planned appointments [79, 88], was identified [31, 40, 50, 79, 84, 89, 93, 100, 105]. In addition, the continued presence of post-reintegration support from a professional who acts as a safety net for the patient to fall back on when struggling return to work or being back at work was characteristic [30, 41, 49, 50, 67, 84, 92, 106–108]. Someone to fall back on was highlighted as a comforting thought [92]. However, when ongoing check-ins are too frequent, patients indicated the follow-ups as being unnecessary and that they experienced a feeling of being put under pressure [71]. Furthermore, to maintain a continuous process, a lack of referral pathways [58, 63, 88, 90, 109], an overload of mandatory paperwork [73], long waiting times [43, 54, 62, 93, 109], and an excessive number of professionals need to be avoided [8, 34, 43, 71, 88, 95, 96, 102, 110].

##### Transparency in the Process Steps

Need for transparency in the multiple process steps, for example by offering a clear overview of the role and responsibility of each professional in the process and clear feedback on how decisions affect the process (CERQual assessment: high confidence).

Transparency in the multiple process steps [63, 89, 94], and clear feedback and reflection on how certain decisions affect the process [64, 79, 84, 93] were highlighted as contributing to good patient expectation management [63, 69, 75, 79, 89, 103, 111]. In addition, patients mentioned a lack of understanding regarding the support and responsibility they could expect from certain professional during the process [75, 77, 87, 92, 94, 105], and some assumed that delivery of work-focused healthcare was outside the realm of the medical specialist [40, 44, 68]. Therefore, the need for information provision regarding the multiple steps in the work-focused healthcare process, such as: what is done, what will happen next, what to expect [79, 96, 100, 110], who is doing what and whose responsibility it is was identified [40, 79, 100].

##### Interdisciplinary Teamwork and Coordination

Need for coherent interaction and constructive collaboration between professionals involved in work-focused healthcare, as well as towards the employer. Involvement of an independent mediator to coordinate the process is suggested (CERQual assessment: high confidence).

Patients experienced a lack of communication between medical, psychological and occupational professionals regarding work issues [54, 63, 75, 97, 108, 110–113], which gave patients the feeling they were acting as their own gatekeeper in the work-focused healthcare process [56, 71, 110, 114]. The lack of communication resulted in inconsistent information and discrepancy in information provision towards patients, causing feelings of confusion, frustration and discouragement [33, 38–41, 72, 110]. In addition, requesting information regarding medical and functional status from the medical system can give patients a feeling of distrust regarding the occupational healthcare professional [111]. Therefore, patients highlighted the importance of coherent interaction and constructive multidisciplinary collaborations between these professionals to facilitate RTW [8, 30, 56, 61, 63, 72, 73, 79, 84, 89, 105, 115], and the need for an independent mediator to coordinate the patient’s process and maintain regular contact between professionals involved [42, 52, 58, 67, 79, 88, 89, 91, 94, 99, 104, 105, 116, 117]. Thereby patients suggested to include occupational healthcare professionals within the multidisciplinary team in clinical care [56].

In addition, in order to put pressure for the advised work modifications to be implemented [44, 46, 75, 83, 109, 118] and to advise and educate the employer about disability management [40, 59, 85, 90–92, 105, 109], communication from work-focused healthcare professionals in the direction of the employer was seen as an important asset [43–46, 79, 83]. Patients stated that when their employer is less supportive, more support by occupational health is needed [32, 49, 85]. On the other hand, the input of the employer’s expectations regarding RTW give the patient the opportunity to highlight the work requirements within the work-focused healthcare process [49, 65].

##### Information About Rights and Regulations

Need for a clear overview of the rights and regulations regarding the work-focused healthcare process and the patient’s obligations, in different formats at multiple time points throughout the process (CERQual assessment: low confidence).

Contradictory or missing information on the legal aspects of the process [31, 70, 78, 110, 116] results in a feeling of distrust [111] and leading to patients fearing they will lose their financial benefits [89]. Therefore, it was indicated that it is important to learn about the legal rights and obligations of both patients and employers with regard to sick-leave and social security shortly after diagnosis [42, 63, 68, 85, 100]. Additionally, patients need practical information on existing regulations [54, 74, 88, 100, 116, 119], e.g. a checklist [88] that can be used as input for certain decisions and taking responsibility in their own process [74, 100]. However, patients indicated that the content of such information [41, 50, 119] and the timing of information provision was often not in line with their needs [49, 63, 111]. It was suggested that information should be provided in diverse formats including verbal and written information at different sources, for example websites, pamphlets, and magazines, as well as at several time points during the healthcare delivery process [49, 73, 100, 111].

#### Supportive Attitude and Behavior

The theme *supportive attitude and behavior* comprises the needs focusing on a positive and supportive attitude and behavior of the healthcare professional that patients encounter throughout their health journey. The subthemes represent the identified needs for: trustful relationship, motivational attitude, equal partnership, and patient advocacy.

##### Trustful Relationship

Need for a trustful relationship with the professional, developed by being treated with respect, taken seriously, being trusted and an emphatic and in-person approach from the professional (CERQual assessment: moderate confidence).

Patients indicated being treated with respect [43, 78, 89, 93, 100, 116], being taken seriously [12, 79, 81, 100–102, 112, 120], being trusted [54, 70, 71, 89, 95, 100, 109, 112, 115, 121], and receiving empathy and compassion [62, 87, 109, 115] from professionals as forming the fundamentals of a trustful relationship. Furthermore, developing a trustful relationship with the patient includes generating a feeling of being welcome, being carefully examined, not being questioned and professionals providing spontaneous information about the process [79, 93, 112]. A trustful relationship strengthens the feeling of being of value to society [79]. However, professionals need to take into account that it takes time to develop a trustful relationship with a patient [116]. In order to provide a feeling of being listened to by the professional, the importance of enough time and in-person consultation was emphasized [38, 40, 54, 55, 57, 60, 71, 75, 79, 89, 101, 112, 115]. In this context, a strict and clear language and attitude of the professional supporting the trust is needed [79].

##### Motivational Attitude

Need for an encouraging, positive, and proactive attitude from professionals, by sharing positive thoughts about the patient’s abilities, to motivate the patient to return to work (CERQual assessment: high confidence).

An encouraging and positive attitude from professionals involved in work-focused healthcare to go back to work is highly appreciated and motivates patients to RTW [33, 35, 36, 40, 41, 44, 59, 62, 78, 79, 112]. Professionals’ advice to not RTW or reduce working hours was experienced as negative by the patients [35, 42, 53, 69, 78]. Therefore, the professional can act as a coach for the patient [119] by providing balanced encouragement [35, 91, 93, 100, 101, 112], providing space to discuss the patient’s fears [55, 93], sharing a positive view on the patient’s abilities [12, 32, 42, 102, 120] and confirming the patient’s thoughts about RTW [39, 40, 76, 87]. Furthermore, a proactive attitude by professionals, taking the initiative in providing solutions and information, was needed [42, 50, 59, 70, 115].

##### Equal Partnership

Need for an equal partnership, with equal power dynamics, between the professional and patient in making decisions regarding vocational reintegration, by listening to and valuing the patient’s choices (CERQual assessment: moderate confidence).

Patients highlighted the need for a relationship with an equal power dynamic in decisions [30, 64, 69, 72, 76, 93, 95, 101, 103, 112, 113], in which they are recognized as equal by the professional [42, 89, 106, 116, 122]. To establish such an equal relationship, the professional needs to listen to, and value the patient’s choices, views and experiences [43, 60, 61, 64, 73, 79, 84, 95, 99, 101, 108, 110, 112, 113, 120]. In other words, the professional should not talk about the patient, but talk with the patient [73, 101, 122]. In addition, to establish equal power dynamics and allowing for shared decision-making, the need for good information provision was mentioned [110]. However, when the patient does not understand how to act, does not have sufficient energy to act, or in other ways needs to be relieved from responsibility in decision-making, it was experienced as a relief when the professional took over [32, 89, 100, 112].

##### Patient Advocacy

Need for the professional to act in the patient’s interests instead of in the interests of other parties, such as the employer (CERQual assessment: moderate confidence).

Representation by occupational healthcare professionals who are affiliated with the company gave the patient mixed feelings about the independent status of these professionals [42, 75, 87]. They mentioned the satisfaction with, and need for, professionals acting in the patient’s interest, instead of being employer-oriented [46, 58, 70, 81, 87, 101, 122].

#### Tailored Approach

The theme *tailored approach* comprises the needs focusing on the delivery of work-focused healthcare tailored to the individuals’ needs. The subthemes represent the identified needs for: flexibility in work-focused healthcare, attention for the personal situation, inclusion of individual goals, and disease-specific information in relation to work.

##### Flexibility in Work-Focused Healthcare

Need for flexibility in the work-focused healthcare provision, and flexibility in the application of the rules in the context of the patient’s needs, in order to receive more tailored support (CERQual assessment: high confidence).

Work-focused encounters were experienced as routine procedures focused on generic protocols and medical aspects, rather than tailored to the patient’s individual needs and capacities [8, 59, 63, 75, 81, 95, 96, 102, 112, 113, 115, 120]. Because of these routine procedures, independent of the patient’s functional abilities, excessive pressure to RTW was experienced by patients [53, 71, 115, 122, 123]. That is why patients stated the need for flexibility in the system in order to receive tailored support and to be treated as a unique individual [39, 42, 47, 48, 62, 64, 70, 85, 100], with a focus on the bigger picture in their everyday life [33, 42, 47, 54, 70, 73, 79, 106] and avoiding excessive pressure [58, 64, 77, 81, 103, 110]. For this, professionals need to apply a flexible approach towards the delivered support [84, 99] and a flexible application of the rules in the context of the patient’s needs [34, 42, 43, 60–62, 71, 78, 93, 96, 102, 107, 110, 112, 116].

##### Attention for the Personal Situation

Need﻿ for attention for the personal situation, including understanding of work capabilities and knowledge of the specific medical situation, on the part of the professional (CERQual assessment: high confidence).

Patients indicated that the experience of not being understood delayed the time to RTW [30, 33, 40, 73, 78, 84]. Therefore, a need for the professional to understand the patient’s personal situation, such as the decreased work capabilities and the related personal needs, and its impact on the patient’s daily life, was identified [74, 81, 86, 89, 91, 99, 100, 121, 122]. This understanding can be established by having conversations with, and listen to the patient [8, 32, 93], and thoroughly read the patient’s files before the start of the consultation [70]. Additionally, knowledge about the specific medical situation, including the physical and psychological impairments, side effects and its complications [8, 35, 39, 42–44, 46, 53, 56, 58, 62, 63, 70, 71, 73, 74, 83, 87, 90, 99, 113, 115, 119] and understanding of the work situation [32, 39, 40, 44, 56, 76, 108, 109] by the professional is crucial for patients to feel understood. In this context, patients indicated confidence in the judgment of their medical specialists, raising their confidence in RTW [49, 76]. To promote understanding of the personal problems with work participation at the workplace, information provision by the occupational healthcare professional towards the colleagues and employer about the consequences of the specific medical condition and individual work-related advice is considered important [12, 35, 49, 62, 63, 69, 84, 88, 90, 94].

##### Inclusion of Patient-Focused Goals

Need for professionals to include patient-focused goals, meeting the patient’s own goals and motivation (CERQual assessment: moderate confidence).

Patients pointed out feeling that the goal of the supporting professional, regarding RTW, support frequency or work tasks, did not always match their own goals [43, 44, 66, 69, 76, 83, 121]. Therefore, patients determined the need for the professional to set patient-focused goals, adjusting the support to their motives to work, their openness to receive guidance [34, 50, 56, 61, 93, 113], and their interest, to achieve a common goal [40, 42, 80, 100].

##### Disease-Specific Information in Relation to Work

Need for information provision on the expected disease-specific consequences on work, such as expected return to work timelines and impact on work-capacity due to the diagnosis (CERQual assessment: high confidence).

A lack of knowledge about the disease, the duration of treatment, potential complications and the influence of these on work made it hard for patients to decide on RTW and to perform effectively while at work [43, 46, 51, 57, 61, 71, 78, 82, 92, 99, 119]. Therefore, a need for more information about disease- and treatment-specific results on work ability, including self-care [77], and disease-specific coping strategies [34, 50, 54, 68, 90, 102, 104, 124], was identified [12, 49, 50, 56, 62, 63, 70, 78, 80, 88, 90, 100], including timelines of expected recovery and impact of side effects on work-capacity over time [46, 51, 65, 68, 70, 71, 98, 111].

## Confidence in the Review Findings

Using the CERQual approach, all identified subthemes (*n* = 17) were assessed for confidence in the representation of the phenomenon of interests. In the quality assessment, nine identified needs (53%) were assessed as high confidence, seven identified needs (41%) as moderate confidence, and one identified need (6%) as low confidence. The main concern identified in the quality assessment was concerning relevance, because a large number of studies representing a small range of geographical, high-income settings. The findings of the assessment with the CERQual approach, including written justification, can be found in the summary of qualitative findings table (Table 2). For insights into the reasoning and explanations behind these assessments for each review finding, see the evidence profile table (online Supplementary Appendix Table 4).

**Table 2:** Summary of qualitative findings table

#	Summarized review finding	GRADE-CERQual assessment of confidence	Explanation of GRADE-CERQual assessment	References
Substantive guidance				
1.1	Work as a topic in healthcare delivery—Need for work-focused support by all professionals throughout the healthcare delivery process, including the medical specialist and rehabilitation professional, to facilitate staying at work or returning to work	High confidence	Minor concerns regarding methodological limitations, No/Very minor concerns regarding coherence, No/Very minor concerns regarding adequacy, and No/Very minor concerns regarding relevance	[30–70]
1.2	Practical and specific guidance—Need to receive practical tips, e.g. on work modifications, and targeted and phased rehabilitation and return to work plans including realistic goals, in order to help the patient avoid exceeding their limits	High confidence	No/Very minor concerns regarding methodological limitations, No/Very minor concerns regarding coherence, No/Very minor concerns regarding adequacy, and Minor concerns regarding relevance	[12, 30, 32, 34, 35, 37, 39, 41, 44, 45, 48–50, 60–62, 68–87]
1.3	Psychological support—Need for psychological assessment and support to help process the impact of the medical condition on impairment in living and working	High confidence	No/Very minor concerns regarding methodological limitations, No/Very minor concerns regarding coherence, No/Very minor concerns regarding adequacy, and Minor concerns regarding relevance	[30–32, 40, 42, 43, 59, 62, 67, 70, 72, 80, 83, 84, 88–93]
1.4	Vocational rehabilitation—Need for vocational rehabilitation to gain insight into and restore functional abilities and to explore suitable work arrangements	Moderate confidence	No/Very minor concerns regarding methodological limitations, Moderate concerns regarding coherence, No/Very minor concerns regarding adequacy, and Minor concerns regarding relevance	[42, 45, 60, 62, 63, 70, 74, 75, 77, 94–97]
Clear and continuous process				
2.1	Early access to support—Need for early presence and access to work-focused healthcare support, by being able to easily reach out and make timely appointments with relevant professionals	Moderate confidence	No/Very minor concerns regarding methodological limitations, Minor concerns regarding coherence, No/Very minor concerns regarding adequacy, and Minor concerns regarding relevance	[30, 31, 42, 43, 45, 46, 59, 60, 63, 70, 71, 73, 75, 77, 79, 84, 87–89, 91, 92, 98–101]
2.2	Continuity in support—Need for continuous work-focused consultations, including continuous presence of support after full return to work, and the option to fall back on someone when struggling with problems with work participation	Moderate confidence	No/Very minor concerns regarding methodological limitations, Minor concerns regarding coherence, No/Very minor concerns regarding adequacy, and Minor concerns regarding relevance	[8, 30, 31, 34, 39–41, 43, 49, 50, 54, 58, 59, 62, 63, 67, 71, 73, 79, 84, 88–90, 92–96, 100, 102–110]
2.3	Transparency in the process steps—Need for transparency in the multiple process steps, for example by offering a clear overview of the role and responsibility of each professional in the process and clear feedback on how decisions affect the process	High confidence	No/Very minor concerns regarding methodological limitations, No/Very minor concerns regarding coherence, No/Very minor concerns regarding adequacy, and Minor concerns regarding relevance	[40, 44, 63, 64, 68, 69, 75, 77, 79, 84, 87, 89, 92–94, 96, 100, 103, 105, 110, 111]
2.4	Interdisciplinary teamwork and coordination—Need for coherent interaction and constructive collaboration between professionals involved in work-focused healthcare, as well as towards the employer. Involvement of an independent mediator to coordinate the process is suggested	High confidence	No/Very minor concerns regarding methodological limitations, No/Very minor concerns regarding coherence, No/Very minor concerns regarding adequacy, and No/Very minor concerns regarding relevance	[8, 30, 32, 33, 38–46, 49, 52, 54, 56, 58, 59, 61, 63, 65, 67, 71–73, 75, 79, 83–85, 88–92, 94, 97, 99, 104, 105, 108–118]
2.5	Information about the rights and regulations—Need for a clear overview of rights and regulations regarding the work-focused healthcare process and the patient’s obligations, in different formats at multiple time points throughout the process	Low confidence	No/Very minor concerns regarding methodological limitations, No/Very minor concerns regarding coherence, No/Very minor concerns regarding adequacy, and Serious concerns regarding relevance	[31, 41, 42, 49, 50, 54, 63, 68, 70, 73, 74, 78, 85, 88, 89, 100, 110, 111, 116, 119]
Supportive attitude and behavior				
3.1	Trustful relationship—Need for a trustful relationship with the professional, developed by being treated with respect, taken seriously, being trusted and an emphatic and in-person approach from the professional	Moderate confidence	No/Very minor concerns regarding methodological limitations, Minor concerns regarding coherence, No/Very minor concerns regarding adequacy, and Minor concerns regarding relevance	[12, 38, 40, 43, 54, 55, 57, 60, 62, 70, 71, 75, 78, 79, 81, 87, 89, 93, 95, 100–102, 109, 112, 115, 116, 120, 121]
3.2	Motivational attitude—Need for an encouraging, positive, and proactive attitude from professionals, by sharing positive thoughts about the patient’s abilities, to motivate the patient to return to work	High confidence	No/Very minor concerns regarding methodological limitations, No/Very minor concerns regarding coherence, No/Very minor concerns regarding adequacy, and Minor concerns regarding relevance	[12, 32, 33, 35, 36, 39–42, 44, 50, 53, 55, 59, 62, 69, 70, 76, 78, 79, 87, 91, 93, 100–102, 112, 115, 119, 120]
3.3	Equal partnership—Need for an equal partnership, with equal power dynamics, between the professional and patient in making decisions regarding vocational reintegration, by listening and valuing the patient’s choices	Moderate confidence	No/Very minor concerns regarding methodological limitations, Minor concerns regarding coherence, No/Very minor concerns regarding adequacy, and Minor concerns regarding relevance	[30, 32, 42, 43, 60, 61, 64, 69, 72, 73, 76, 79, 84, 89, 93, 95, 99–101, 106, 108, 110, 112, 113, 116, 120, 122]
3.4	Patient advocacy—Need for the professional to act in the patient’s interest instead of the interests of other parties, such as the employer	Moderate confidence	Minor concerns regarding methodological limitations, No/Very minor concerns regarding coherence, Moderate concerns regarding adequacy, and No/Very minor concerns regarding relevance	[42, 46, 58, 70, 75, 81, 87, 100, 101, 122]
Tailored approach				
4.1	Flexibility in work-focused healthcare—Need for flexibility in the work-focused healthcare provision, and flexibility in the application of the rules in the context of the patient’s needs, in order to receive more tailored support	High confidence	No/Very minor concerns regarding methodological limitations, No/Very minor concerns regarding coherence, Minor concerns regarding adequacy, and Minor concerns regarding relevance	[8, 33, 34, 39, 42, 43, 47, 48, 53, 54, 58–64, 70, 71, 73, 75, 77–79, 81, 84, 85, 93, 95, 96, 99, 100, 102, 103, 106, 107, 110, 112, 113, 115, 116, 120, 122, 123]
4.2	Attention for the personal situation—Need for attention for the personal situation, including understanding of work capabilities and knowledge of the specific medical situation, on the part of the professional	High confidence	No/Very minor concerns regarding methodological limitations, No/Very minor concerns regarding coherence, No/Very minor concerns regarding adequacy, and Minor concerns regarding relevance	[8, 12, 30, 32, 33, 35, 39, 40, 42–44, 46, 49, 53, 56, 58, 62, 63, 69–71, 73, 74, 76, 78, 79, 81, 83, 84, 86–91, 93, 94, 99, 100, 108, 109, 113, 115, 119, 121, 122]
4.3	Inclusion of patient-focused goals—Need for professionals to include patient-focused goals, meeting the patient’s own goals and motivation	Moderate confidence	No/Very minor concerns regarding methodological limitations, No/Very minor concerns regarding coherence, Minor concerns regarding adequacy, and Minor concerns regarding relevance	[34, 40, 42–44, 50, 56, 61, 66, 69, 76, 80, 83, 93, 100, 113, 121]
4.4	Disease-specific information in relation to work—Need for information provision on the expected disease-specific consequences on work, such as expected return to work timelines and impact on work-capacity due to the diagnosis	High confidence	No/Very minor concerns regarding methodological limitations, No/Very minor concerns regarding coherence, No/Very minor concerns regarding adequacy, and No/Very minor concerns regarding relevance	[12, 34, 43, 46, 49–51, 54, 56, 57, 61–63, 65, 68, 70, 71, 77, 78, 80, 82, 88, 90, 92, 98–100, 102, 104, 111, 119, 124]

## Discussion

### Summary of Main Findings

This qualitative evidence synthesis included 97 studies representing perspectives on work-focused healthcare from patients with varied chronic medical conditions in different work settings. We identified a wide range of patients’ needs regarding work-focused healthcare provided by various healthcare professionals (*n* = 17), categorized into four main themes: 1. substantive guidance, 2. clear and continuous process, 3. supportive attitude and behavior, and 4. tailored approach. Overall, the confidence in the identified needs was rated moderate to high using the CERQual approach, which makes it highly likely that the review findings are a reasonable representation of patients’ needs regarding work-focused healthcare when experiencing problems with work participation due to a chronic medical condition.

### Agreements and Disagreements with Other Studies or Reviews

In accordance with the patient perspective as highlighted in this review, earlier studies show that healthcare professionals also agree that work is an important outcome for health and wellbeing [125]. In addition, patient-centred healthcare delivery has been found to increase patient satisfaction [126]. However, healthcare professionals acknowledge that actual provision of patient-centred work-focused healthcare is often limited [125, 127]. Supporting evidence-based medicine interventions is considered important by healthcare professionals to improve their patient-centred work-focused healthcare delivery [128].

Some of our findings, including long waiting times for referrals, difficult access to consultations, and poor long-term support, have also been identified as common barriers for proper care delivery in the curative care from patients’ perspective [129]. Earlier studies found that healthcare professionals identified a lack of communication with other professionals as a barrier for patient-centred care [130]. In line with our review findings, the need for accessible care and good information provision regarding the care process were previously identified in primary and curative healthcare [131].

Furthermore, earlier research, as well as findings from the current study, shows that problems with work participation may vary between individuals, emphasizing the importance of tailored work-focused healthcare [132]. Aligning with our current study findings, earlier studies in curative care report the need for an individualized, flexible, and holistic relationship with the healthcare professional, who is familiar with the patient’s specific medical conditions and their goals [129, 131]. In a work-focused healthcare setting, our findings also show that patients require a tailored approach that is sensitive to the patient’s situation and needs. Our findings support shared decision-making as an approach that could be explored in work-focused healthcare delivery by tailoring care to the patient’s individual needs [133], while supporting an equal partnership between the patient and professional [134–136].

In earlier research, multiple strategies have been described to enhance communication between professionals involved in work-focused healthcare, for example by implementing a protocol or a communication form [128, 137, 138]. As also considered true in the findings of the current study, interdisciplinary teamwork between professionals may improve not only promote a clear and continuous care process, but may also increase trust and commitment levels of patients in the process [139]. A trustful relationship and equal partnership between the patient and professional, which is found to be an important need in the context of the current study, is also considered important by both professionals and patients regardless of whether the healthcare setting is focused on work or not [140, 141]. Supporting our review findings, empathy, as the basis of a trustful relationship between the professional and patient, needs to consist of understanding the personal situation of the patient, and communicating this understanding to the patient in a supportive way [142]. Moreover, in accordance with this review, it is suggested that a motivational attitude on the part of the healthcare professional towards the patient may assist in patients’ behavioral changes, patients’ autonomy and fulfillment of patient-centred goals [143].

### Strengths and Limitations

The strengths of the current qualitative evidence synthesis lie in its extensive search across multiple databases, large number of studies included, and broad target population, enhancing the generalizability of the findings. Methodologically, the use of the CERQual approach, which aligns with international recommendations [23], ensures transparency in the confidence of the findings [25]. In addition, consensus meetings between authors further improved the trustworthiness of our evidence synthesis.

Nevertheless, there were also some methodological limitations in the current qualitative evidence synthesis. Although the pragmatic decision was made to only include studies published in the English language, we may have excluded relevant literature from other perspectives in other languages. Nonetheless, given the large number of studies and countries included, the impact of this language restriction may be limited [144]. Moreover, as indicated in the assessment by the CERQual approach, the majority of the included studies were conducted in high-income countries where workers typically have stronger social security regulations. This dominance may limit the generalizability of our findings to healthcare systems from low- or middle income countries where workers may receive lower levels of work-related protection and support.

### Implications for Practice

New strategies are needed to realize patient-centred work-focused healthcare. The needs from the patient’s perspective, as reflected on in this qualitative evidence synthesis, provide the starting point for policy makers and (occupational) healthcare professionals to change current practice to achieve better patient-centred work-focused healthcare. In addition, to assess the success of such innovations, patient-centred outcomes should be monitored within work-focused healthcare [145].

Moreover, the broader understanding of patients’ needs in work-focused healthcare can help (occupational) healthcare professionals adopt a more patient-centred approach in practice. Professionals can assess their patient-centredness using the identified needs as a checklist, guideline or communication tool during consultations.

### Implications for Research

In this evidence synthesis, we showed a considerable number of needs that fit the aims of work-focused healthcare in patients with a chronic disease. It may be relevant to explore whether these needs vary in intensity or priority in different subgroups, for example different types of diseases and workplace characteristics. In addition, as the timing of care may influence patients’ needs within the work-focused healthcare provision [146], we suggest that future qualitative studies should consider the time and place of care delivery within the individual patient trajectories during the work-focused healthcare process. Exploring the intensity or priority of the needs identified in this study among different subgroups or at different time points may enhance theory development in the future.

In addition, the need for *information about the rights and regulations* was assessed with low confidence due to serious concerns regarding relevance, thereby questioning the timing and form of this information provision. Other studies identified the need for more clarity regarding the rights and regulations among professionals involved in work-focused healthcare [147]. Therefore, future research needs to assess the requirements for education on rights and regulations in work-focused healthcare for all stakeholder groups.

## Conclusion

This review identified four main themes, representing 17 subthemes, containing needs regarding work-focused healthcare from a broad patient population. Increasing insight into patients’ needs in work-focused healthcare can guide policymakers and (occupational) healthcare professionals in developing new intervention and care strategies important for patient-centred work-focused healthcare. Future research should investigate whether the intensity or priority of the needs identified in this study varies among different subgroups or at different time points. Insight is also needed into what these new strategies should consist of.

## Supplementary Information

Below is the link to the electronic supplementary material.

Supplementary file1 (DOCX 353 KB)

## Data Availability

The datasets generated during and/or analysed during the current study are available from the corresponding author on reasonable request.

## References

[1] European Commission. Population aging in Europe: facts, implications and policies. Outcomes of EU-funded research. 2014. https://op.europa.eu/en/publication-detail/-/publication/1e7549b4-2333-413b-972c-f9f1bc70d4cf1

[2] Van Solinge H, Henkens K. Older workers’ emotional reactions to rising retirement age: the case of the Netherlands. Work, Aging Retire. 2017;3(3):273–283.

[3] Rabate S, Rochut J. Employment and substitution effects of raising the statutory retirement age in France. J Pension Econ Finance. 2020;19(3):293–308.

[4] WHO. Ageing and health. Available from: https://www.who.int/news-room/fact-sheets/detail/ageing-and-health.

[5] Lagerveld SE, Bültmann U, Franche R-L, Van Dijk F, Vlasveld MC, van der Feltz-Cornelis CM, et al. Factors associated with work participation and work functioning in depressed workers: a systematic review. J Occup Rehabil. 2010;20(3):275–292. 20091105 10.1007/s10926-009-9224-x PMC2923705

[6] Vooijs M, Leensen MC, Hoving JL, Daams JG, Wind H, Frings-Dresen MH. Disease-generic factors of work participation of workers with a chronic disease: a systematic review. Int Arch Occup Environ Health. 2015;88(8):1015–1029. 25712761 10.1007/s00420-015-1025-2 PMC4608993

[7] Bernklev T, Jahnsen J, Henriksen M, Lygren I, Aadland E, Sauar J, et al. Relationship between sick leave, unemployment, disability, and health-related quality of life in patients with inflammatory bowel disease. Inflamm Bowel Dis. 2006;12(5):402–412. 16670530 10.1097/01.MIB.0000218762.61217.4a

[8] Juurlink TT, Vukadin M, Stringer B, Westerman MJ, Lamers F, Anema JR, et al. Barriers and facilitators to employment in borderline personality disorder: a qualitative study among patients, mental health practitioners and insurance physicians. PLoS ONE. 2019;14(7): e0220233. 31335909 10.1371/journal.pone.0220233 PMC6650068

[9] Mbengi RK, Otter R, Mortelmans K, Arbyn M, Van Oyen H, Bouland C, et al. Barriers and opportunities for return-to-work of cancer survivors: time for action—rapid review and expert consultation. Syst Rev. 2016;5(1):1–10. 26912175 10.1186/s13643-016-0210-z PMC4765094

[10] Dekkers-Sánchez PM, Wind H, Sluiter JK, Frings-Dresen MH. A qualitative study of perpetuating factors for long-term sick leave and promoting factors for return to work: chronic work disabled patients in their own words. J Rehabil Med. 2010;42(6):544–552. 20549159 10.2340/16501977-0544

[11] Bartys S, Stochkendahl MJ. Section 10, Chapter 12: Work-focused healthcare for low back pain. In: Scott D, Boden MD, editors. International society for the study of the lumbar spine online textbook; 2018. https://www.wheelessonline.com/ISSLS/section-10-chapter-12-work-focused-healthcare-for-low-back-pain

[12] Rubenson C, Svensson E, Linddahl I, Björklund A. Experiences of returning to work after acquired brain injury. Scand J Occup Ther. 2007;14(4):205–214. 18236320 10.1080/11038120601110934

[13] Bertakis KD, Azari R. Patient-centered care is associated with decreased health care utilization. J Am Board Family Med. 2011;24(3):229–239. 10.3122/jabfm.2011.03.100170 21551394

[14] Lee T, Porter M. The strategy that will fix healthcare. Boston: Harvard Business Review; 2013

[15] Corrigan JM. Crossing the quality chasm. In: Reid PR, Compton WD, Grossman JH, et al. Building a better delivery system: A new engineering/healthcare partnership. National academies press; 2005. p. 95–97. http://www.nap.edu/catalog/11378.html

[16] Sevin C, Moore G, Shepherd J, Jacobs T, Hupke C. Transforming care teams to provide the best possible patient-centered, collaborative care. J Ambul Care Manag. 2009;32(1):24–31. 10.1097/01.JAC.0000343121.07844.e0 19104291

[17] Tong A, Flemming K, McInnes E, Oliver S, Craig J. Enhancing transparency in reporting the synthesis of qualitative research: ENTREQ. BMC Med Res Methodol. 2012;12(1):1–8. 23185978 10.1186/1471-2288-12-181 PMC3552766

[18] Lefebvre C, Glanville J, Briscoe S, Littlewood A, Marshall C, Metzendorf M-I, et al. Chapter 4: Searching for and selecting studies. In: Higgins JPT, Thomas J, Chandler J, Cumpston M, Li T, Page MJ, Welch VA (editors) Cochrane handbook for systematic reviews of interventions version 62 (updated February 2021) 2021.

[19] Ouzzani M, Hammady H, Fedorowicz Z, Elmagarmid A. Rayyan—a web and mobile app for systematic reviews. Syst Rev. 2016;5:210. 27919275 10.1186/s13643-016-0384-4 PMC5139140

[20] Thomas J, Harden A. Methods for the thematic synthesis of qualitative research in systematic reviews. BMC Med Res Methodol. 2008;8(1):1–10. 18616818 10.1186/1471-2288-8-45 PMC2478656

[21] CASP. Checklist: 10 questions to help you make sense of a qualitative research. 2018 [Available from: CASP-Qualitative-Checklist-2018.pdf (casp-uk.net).

[22] Kanavaki AM, Rushton A, Klocke R, Abhishek A, Duda JL. Barriers and facilitators to physical activity in people with hip or knee osteoarthritis: protocol for a systematic review of qualitative evidence. BMJ Open. 2016;6(11): e012049. 27810971 10.1136/bmjopen-2016-012049 PMC5128852

[23] Lewin S, Glenton C, Munthe-Kaas H, Carlsen B, Colvin CJ, Gülmezoglu M, et al. Using qualitative evidence in decision making for health and social interventions: an approach to assess confidence in findings from qualitative evidence syntheses (GRADE-CERQual). PLoS Med. 2015;12(10): e1001895. 26506244 10.1371/journal.pmed.1001895 PMC4624425

[24] GRADE-CERQual Interactive Summary of Qualitative Findings (iSoQ) [Computer program]. Version 1.0 accessed [March 2022]. Oslo, Norway: Norwegian Institute of Public Health (developed by the Epistemonikos Foundation, Megan Wainwright Consulting and the Norwegian Institute of Public Health for the GRADE-CERQual Project Group). Available at isoq.epistemonikos.org.

[25] Lewin S, Booth A, Glenton C, Munthe-Kaas H, Rashidian A, Wainwright M, et al. Applying GRADE-CERQual to qualitative evidence synthesis findings: introduction to the series. BioMed Central. 2018;13:1–10. 10.1186/s13012-017-0688-3 PMC5791040 29384079

[26] Munthe-Kaas H, Bohren MA, Glenton C, Lewin S, Noyes J, Tunçalp Ö, et al. Applying GRADE-CERQual to qualitative evidence synthesis findings—paper 3: how to assess methodological limitations. Implement Sci. 2018;13(1):25–32. 29384078 10.1186/s13012-017-0690-9 PMC5791044

[27] Colvin CJ, Garside R, Wainwright M, Munthe-Kaas H, Glenton C, Bohren MA, et al. Applying GRADE-CERQual to qualitative evidence synthesis findings—paper 4: how to assess coherence. Implement Sci. 2018;13(1):33–41. 29384081 10.1186/s13012-017-0691-8 PMC5791039

[28] Glenton C, Carlsen B, Lewin S, Munthe-Kaas H, Colvin CJ, Tunçalp Ö, et al. Applying GRADE-CERQual to qualitative evidence synthesis findings—paper 5: how to assess adequacy of data. Implement Sci. 2018;13(1):43–50. 29384077 10.1186/s13012-017-0692-7 PMC5791045

[29] Noyes J, Booth A, Lewin S, Carlsen B, Glenton C, Colvin CJ, et al. Applying GRADE-CERQual to qualitative evidence synthesis findings–paper 6: how to assess relevance of the data. Implement Sci. 2018;13(1):51–61. 29384080 10.1186/s13012-017-0693-6 PMC5791042

[30] Hooson JM, Coetzer R, Stew G, Moore A. Patients’ experience of return to work rehabilitation following traumatic brain injury: a phenomenological study. Neuropsychol Rehabil. 2013;23(1):19–44. 22905786 10.1080/09602011.2012.713314

[31] Jansson I, Björklund A. The experience of returning to work. Work. 2007;28(2):121–134. 17312344

[32] Libeson L, Downing M, Ross P, Ponsford J. The experience of return to work in individuals with traumatic brain injury (TBI): a qualitative study. Neuropsychol Rehabil. 2020;30(3):412–429. 29745289 10.1080/09602011.2018.1470987

[33] Lork K, Holmgren K. The experience of return to work self-efficacy among people on sick leave. Work. 2018;59(4):479–490. 29733041 10.3233/WOR-182697

[34] Audhoe SS, Nieuwenhuijsen K, Hoving JL, Sluiter JK, Frings-Dresen MH. Perspectives of unemployed workers with mental health problems: barriers to and solutions for return to work. Disabil Rehabil. 2018;40(1):28–34. 27756177 10.1080/09638288.2016.1242170

[35] Tamminga SJ, De Boer AG, Verbeek JH, Frings-Dresen MH. Breast cancer survivors’ views of factors that influence the return-to-work process-a qualitative study. Scand J Work, Environ Health. 2012;38:144–154. 21986836 10.5271/sjweh.3199

[36] Dewa CS, Trojanowski L, Tamminga SJ, Ringash J, McQuestion M, Hoch JS. Work-related experiences of head and neck cancer survivors: an exploratory and descriptive qualitative study. Disabil Rehabil. 2018;40(11):1252–1258. 28637151 10.1080/09638288.2017.1291764

[37] Amir Z, Neary D, Luker K. Cancer survivors’ views of work 3 years post diagnosis: a UK perspective. Eur J Oncol Nurs. 2008;12(3):190–197. 18342571 10.1016/j.ejon.2008.01.006

[38] Bae KR, Cho J. Changes after cancer diagnosis and return to work: experience of Korean cancer patients. BMC Cancer. 2021;21(1):1–11. 33478405 10.1186/s12885-021-07812-w PMC7818925

[39] Bardgett M, Lally J, Malviya A, Deehan D. Return to work after knee replacement: a qualitative study of patient experiences. BMJ Open. 2016;6(2): e007912. 26832426 10.1136/bmjopen-2015-007912 PMC4746460

[40] Berger I, Beck L, Jones J, MacEachen E, Kirsh B. Exploring the needs of cancer survivors when returning to or staying in the workforce. J Occup Rehabil. 2020;30(3):480–495. 32016649 10.1007/s10926-020-09877-z

[41] Blokzijl F, Onrust M, Dieperink W, Keus F, van der Horst IC, Paans W, et al. Barriers that obstruct return to work after coronary bypass surgery: a qualitative study. J Occup Rehabil. 2021;31(2):316–322. 32803466 10.1007/s10926-020-09919-6 PMC8172483

[42] Bosma A, Boot C, Schaafsma F, Anema J. Facilitators, barriers and support needs for staying at work with a chronic condition: a focus group study. BMC Public Health. 2020;20(1):1–11. 32033556 10.1186/s12889-020-8320-x PMC7006125

[43] Brakenridge CL, Leow CKL, Kendall M, Turner B, Valiant D, Quinn R, et al. Exploring the lived return-to-work experience of individuals with acquired brain injury: use of vocational services and environmental, personal and injury-related influences. Disabil Rehabil. 2021;44:1–11. 10.1080/09638288.2021.1903101 33794118

[44] Coole C, Watson PJ, Drummond A. Staying at work with back pain: patients’ experiences of work-related help received from GPs and other clinicians: a qualitative study. BMC Musculoskelet Disord. 2010;11(1):1–7. 20799938 10.1186/1471-2474-11-190 PMC2936348

[45] Hartke RJ, Trierweiler R, Bode R. Critical factors related to return to work after stroke: a qualitative study. Top Stroke Rehabil. 2011;18(4):341–351. 21914598 10.1310/tsr1804-341

[46] Jain A, Aggarwal A, Adams J, Jordan RE, Sadhra S, Dubey S, et al. Work productivity loss among rheumatoid arthritis patients in India: a qualitative study. Rheumatol Adv Pract. 2019. 10.1093/rap/rkz046. 32016165 10.1093/rap/rkz046 PMC6988515

[47] Kennedy F, Haslam C, Munir F, Pryce J. Returning to work following cancer: a qualitative exploratory study into the experience of returning to work following cancer. Eur J Cancer Care. 2007;16(1):17–25. 10.1111/j.1365-2354.2007.00729.x 17227349

[48] Lindahl M, Hvalsoe B, Poulsen JR, Langberg H. Quality in rehabilitation after a working age person has sustained a fracture: partnership contributes to continuity. Work. 2013;44(2):177–189. 23324675 10.3233/WOR-121498

[49] Nouri F, Coole C, Baker P, Drummond A. Return to work advice after total hip and knee replacement. Occup Med. 2020;70(2):113–118. 10.1093/occmed/kqaa014 32009167

[50] Van der Meer M, Hoving JL, Vermeulen MI, Herenius MM, Tak PP, Sluiter JK, et al. Experiences and needs for work participation in employees with rheumatoid arthritis treated with anti-tumour necrosis factor therapy. Disabil Rehabil. 2011;33(25–26):2587–2595. 21671833 10.3109/09638288.2011.582923

[51] Zamanzadeh V, Valizadeh L, Rahmani A, Zirak M, Desiron H. Cancer survivors’ experiences of return to work: a qualitative study. Psychooncology. 2018;27(10):2398–2404. 30030874 10.1002/pon.4840

[52] Beerda DC, Zegers AD, van Andel ES, Becker-Commissaris A, van der Vorst MJ, Tange D, et al. Experiences and perspectives of patients with advanced cancer regarding work resumption and work retention: a qualitative interview study. Support Care Cancer. 2022;30(12):9713–9721. 36434411 10.1007/s00520-022-07436-1 PMC9702727

[53] Bennink C, van Der Klift M, Scheurer H, Sonneveld P, Duijts SF. Perspectives on returning to work of multiple myeloma patients: a qualitative interview study. Eur J Cancer Care. 2021;30(6): e13481. 10.1111/ecc.13481 PMC9285059 34152665

[54] Kluit L, de Wind A, Oosting IJ, van Velzen JM, Beumer A, Sluman MA, et al. Current practices, needs, and expectations of discussing work with a medical specialist from a patient’s perspective: a qualitative study. Disabil Rehabil. 2022; 46(1):115–128. 10.1080/09638288.2022.2157500. 36564948 10.1080/09638288.2022.2157500

[55] Miller A, Wilson E, Diver C. Returning to work: a qualitative study of the experiences of head and neck cancer survivors. J Laryngol Otol. 2023;137(6):691–696. 36999532 10.1017/S0022215122002201

[56] Oosting IJ, Kluit L, Schaafsma FG, Beumer A, van Bennekom CA, de Boer AG, et al. Patients’ experiences, needs, and expectations of cooperation between medical specialists and occupational health physicians: a qualitative study. J Occup Environ Med. 2023;65(6): e395. 36882873 10.1097/JOM.0000000000002833 PMC10227924

[57] Pahlplatz T, Schafroth M, Krijger C, Hylkema T, van Dijk C, Frings-Dresen M, et al. Beneficial and limiting factors in return to work after primary total knee replacement: patients’ perspective. Work. 2021;69(3):895–902. 34180460 10.3233/WOR-213522 PMC8385499

[58] Urquhart R, Scruton S, Kendell C. Understanding cancer survivors’ needs and experiences returning to work post-treatment: a longitudinal qualitative study. Curr Oncol. 2022;29(5):3013–3025. 35621635 10.3390/curroncol29050245 PMC9139703

[59] Lock S, Jordan L, Bryan K, Maxim J. Work after stroke: focusing on barriers and enablers. Disabil Soc. 2005;20(1):33–47.

[60] Maillette P, Coutu M-F, Gaudreault N. Workers’ perspectives on return to work after total knee arthroplasty. Ann Phys Rehabil Med. 2017;60(5):299–305. 28347691 10.1016/j.rehab.2017.01.004

[61] Medin J, Barajas J, Ekberg K. Stroke patients’ experiences of return to work. Disabil Rehabil. 2006;28(17):1051–1060. 16950735 10.1080/09638280500494819

[62] Öster C, Kildal M, Ekselius L. Return to work after burn injury: burn-injured individuals’ perception of barriers and facilitators. J Burn Care Res. 2010;31(4):540–550. 20616648 10.1097/BCR.0b013e3181e4d692

[63] Watter K, Kennedy A, McLennan V, Vogler J, Jeffery S, Murray A, et al. Consumer perspectives of vocational rehabilitation and return to work following acquired brain injury. Brain Impairment. 2021;23:164–184. 10.1017/BrImp.2021.4.

[64] Corbière M, Charette-Dussault É, Larivière N. Recognition during the return-to-work process in workers with common mental disorders. J Occup Rehabil. 2022;33:486–505. 10.1007/s10926-022-10087-y. 36462069 10.1007/s10926-022-10087-y

[65] Lysaght RM, Larmour-Trode S. An exploration of social support as a factor in the return-to-work process. Work. 2008;30(3):255–266. 18525149

[66] Ryan CG, Lauchlan D, Rooney L, Hollins Martins C, Gray H. Returning to work after long term sickness absence due to low back pain–the struggle within: a qualitative study of the patient’s experience. Work. 2014;49(3):433–444. 23787255 10.3233/WOR-131646

[67] Karcz K, Schiffmann B, Schwegler U, Staubli S, Finger ME. Facilitators and barriers to sustainable employment after spinal cord injury or acquired brain injury: the person’s perspective. Front Rehabil Sci. 2022;3: 872782. 36188977 10.3389/fresc.2022.872782 PMC9397900

[68] Frazier LM, Miller VA, Miller BE, Horbelt DV, Delmore JE, Ahlers-Schmidt CR. Cancer-related tasks involving employment: opportunities for clinical assistance. J Support Oncol. 2009;7(6):229. 20380331 PMC2855160

[69] Gilworth G, Phil M, Cert A, Sansam K, Kent R. Personal experiences of returning to work following stroke: an exploratory study. Work. 2009;34(1):95–103. 19923680 10.3233/WOR-2009-0906

[70] Vooijs M, Leensen MC, Hoving JL, Wind H, Frings-Dresen MH. Perspectives of people with a chronic disease on participating in work: a focus group study. J Occup Rehabil. 2017;27(4):593–600. 28101790 10.1007/s10926-016-9694-6 PMC5709457

[71] Graff HJ, Deleu NW, Christiansen P, Rytter HM. Facilitators of and barriers to return to work after mild traumatic brain injury: a thematic analysis. Neuropsychol Rehabil. 2021;31(9):1349–1373. 32584206 10.1080/09602011.2020.1778489

[72] MacLennan SJ, Cox T, Murdoch S, Eatough V. An interpretative phenomenological analysis of the meaning of work to women living with breast cancer. Chronic Illn. 2021;18(3):503–516. 10.1177/1742395320987883. 33475434 10.1177/1742395320987883

[73] Netto JA, Yeung P, Cocks E, McNamara B. Facilitators and barriers to employment for people with mental illness: a qualitative study. J Vocat Rehabil. 2016;44(1):61–72.

[74] Decuman S, Smith V, Grypdonck M, De Keyser F, Verhaeghe S. Factors influencing the occupational trajectory of patients with systemic sclerosis: a qualitative study. Clin Exp Rheumatol. 2015;33(Suppl 91):S26–S30. 25797634

[75] Coole C, Watson PJ, Drummond A. Low back pain patients’ experiences of work modifications; a qualitative study. BMC Musculoskelet Disord. 2010;11(1):1–10. 21134248 10.1186/1471-2474-11-277 PMC3016306

[76] Newington L, Brooks C, Warwick D, Adams J, Walker-Bone K. Return to work after carpal tunnel release surgery: a qualitative interview study. BMC Musculoskelet Disord. 2019;20(1):1–11. 31113433 10.1186/s12891-019-2638-5 PMC6530142

[77] Hjärtström C, Norberg AL, Johansson G, Bodin T. To work despite chronic health conditions: a qualitative study of workers at the Swedish public employment service. BMJ Open. 2018;8(4): e019747. 29678972 10.1136/bmjopen-2017-019747 PMC5914773

[78] Nilsson M, Olsson M, Wennman-Larsen A, Petersson L-M, Alexanderson K. Return to work after breast cancer: women’s experiences of encounters with different stakeholders. Eur J Oncol Nurs. 2011;15(3):267–274. 21478053 10.1016/j.ejon.2011.03.005

[79] Sturesson M, Edlund C, Falkdal AH, Bernspång B. Healthcare encounters and return to work: a qualitative study on sick-listed patients’ experiences. Primary Health Care Res Dev. 2014;15(4):464–475. 10.1017/S1463423614000255 25098326

[80] Bridger K, Kellezi B, Kendrick D, Radford K, Timmons S, Rennoldson M, et al. Patient perspectives on key outcomes for vocational rehabilitation interventions following traumatic injury. Int J Environ Res Public Health. 2021;18(4):2035. 33669750 10.3390/ijerph18042035 PMC7922329

[81] Dorland H, Abma F, Roelen C, Smink J, Ranchor A, Bültmann U. Factors influencing work functioning after cancer diagnosis: a focus group study with cancer survivors and occupational health professionals. Support Care Cancer. 2016;24(1):261–266. 26022706 10.1007/s00520-015-2764-z PMC4669376

[82] Gilworth G, Eyres S, Carey A, Bhakta B, Tennant A. Working with a brain injury: personal experiences of returning to work following a mild or moderate brain injury. J Rehabil Med. 2008;40(5):334–339. 18461257 10.2340/16501977-0169

[83] Mansfield E, Stergiou-Kita M, Kirsh B, Colantonio A. After the storm: the social relations of return to work following electrical injury. Qual Health Res. 2014;24(9):1183–1197. 25097188 10.1177/1049732314545887

[84] McRae P, Hallab L, Simpson G. Navigating employment pathways and supports following brain injury in Australia: client perspectives. Australian J Rehabil Couns. 2016;22(2):76–92.

[85] Knott V, Zrim S, Shanahan EM, Anastassiadis P, Lawn S, Kichenadasse G, et al. Returning to work following curative chemotherapy: a qualitative study of return to work barriers and preferences for intervention. Support Care Cancer. 2014;22(12):3263–3273. 25066834 10.1007/s00520-014-2324-y

[86] Beaulieu K. Lived experiences of return to paid work following a brain injury. Br J Occup Ther. 2019;82(11):658–665.

[87] Zaman A, Bruinvels D, de Boer A, Frings-Dresen M. Supporting cancer patients with work-related problems through an oncological occupational physician: a feasibility study. Eur J Cancer Care. 2017;26(5): e12378. 10.1111/ecc.12378 26332251

[88] Gard G, Pessah-Rasmussen H, Brogårdh C, Nilsson Å, Lindgren I. Need for structured healthcare organization and support for return to work after stroke in Sweden: experiences of stroke survivors. J Rehabil Med. 2019;51(10):741–748. 31468058 10.2340/16501977-2591

[89] Hubertsson J, Petersson IF, Arvidsson B, Thorstensson CA. Sickness absence in musculoskeletal disorders-patients’ experiences of interactions with the social insurance agency and health care: a qualitative study. BMC Public Health. 2011;11(1):1–9. 21324175 10.1186/1471-2458-11-107 PMC3050746

[90] Klaver KM, Duijts SF, Engelhardt EG, Geusgens CA, Aarts MJ, Ponds RW, et al. Cancer-related cognitive problems at work: experiences of survivors and professionals. J Cancer Surviv. 2020;14(2):168–178. 31768861 10.1007/s11764-019-00830-5 PMC7182611

[91] Noordik E, Nieuwenhuijsen K, Varekamp I, van der Klink JJ, van Dijk JF. Exploring the return-to-work process for workers partially returned to work and partially on long-term sick leave due to common mental disorders: a qualitative study. Disabil Rehabil. 2011;33(17–18):1625–1635. 21171843 10.3109/09638288.2010.541547

[92] Yarker J, Munir F, Bains M, Kalawsky K, Haslam C. The role of communication and support in return to work following cancer-related absence. Psychooncology. 2010;19(10):1078–1085. 20014202 10.1002/pon.1662

[93] Pasanen J. The nature of positive encounters between disabled workers and insurers in the return to work process. Work. 2021;70(1):287–300. 34511472 10.3233/WOR-213573

[94] Hellman T, Bergström A, Eriksson G, Hansen Falkdal A, Johansson U. Return to work after stroke: important aspects shared and contrasted by five stakeholder groups. Work. 2016;55(4):901–911. 28059820 10.3233/WOR-162455

[95] Wallstedt-Paulsson E, Erlandsson LK, Eklund M. Client experiences in work rehabilitation in Sweden: a one-year follow-up study. Occup Ther Int. 2007;14(1):28–41. 17623377 10.1002/oti.223

[96] Holmlund L, Guidetti S, Eriksson G, Asaba E. Return to work in the context of everyday life 7–11 years after spinal cord injury—a follow-up study. Disabil Rehabil. 2018;40(24):2875–2883. 28793801 10.1080/09638288.2017.1362597

[97] Aguiar-Fernández F, Rodríguez-Castro Y, Botija M, Martínez-Román R. Experiences of female breast cancer survivors concerning their return to work in Spain. Behav Sci. 2021;11(10):135. 34677228 10.3390/bs11100135 PMC8533326

[98] Österholm JH, Björk M, Håkansson C. Factors of importance for maintaining work as perceived by men with arthritis. Work. 2013;45(4):439–448. 23241708 10.3233/WOR-121542

[99] Olischläger DL, den Boer LXY, de Heus E, Brom L, Dona DJ, Klümpen H-J, et al. Rare cancer and return to work: experiences and needs of patients and (health care) professionals. Disabil Rehabil. 2022;45(16):2585–2596. 10.1080/09638288.2022.2099589. 35850601 10.1080/09638288.2022.2099589

[100] Mårtensson L, Hensing G. Experiences of factors contributing to women’s ability to make informed decisions about the process of rehabilitation and return to work: a focus group study. Work. 2012;43(2):237–248. 22927623 10.3233/WOR-2012-1397

[101] Mussener U, Svensson T, Soderberg E, Alexanderson K. Encouraging encounters: sick-listed persons’ experiences of interactions with rehabilitation professionals. Soc Work Health Care. 2007;46(2):71–87. 10.1300/j010v46n02_05 18192198

[102] Van Egmond M, Duijts S, Loyen A, Vermeulen S, Van der Beek A, Anema J. Barriers and facilitators for return to work in cancer survivors with job loss experience: a focus group study. Eur J Cancer Care. 2017;26(5): e12420. 10.1111/ecc.12420 PMC5600095 26603683

[103] Joosen MC, Lugtenberg M, Arends I, van Gestel HJ, Schaapveld B, Terluin B, et al. Barriers and facilitators for return to work from the perspective of workers with common mental disorders with short, medium and long-term sickness absence: a longitudinal qualitative study. J Occup Rehabil. 2021;32:272–283. 10.1007/s10926-021-10004-9. 34580811 10.1007/s10926-021-10004-9 PMC9232415

[104] Madsen CMT, Christensen JR, Bremander A, Primdahl J. Perceived challenges at work and need for professional support among people with inflammatory arthritis-a qualitative interview study. Scand J Occup Ther. 2021;30(5):640–649. 10.1080/11038128.2021.1989483. 34644224 10.1080/11038128.2021.1989483

[105] Holmlund L, Hellman T, Engblom M, Kwak L, Sandman L, Törnkvist L, et al. Coordination of return-to-work for employees on sick leave due to common mental disorders: facilitators and barriers. Disabil Rehabil. 2020;44(13):3113–3121. 10.1080/09638288.2020.1855263. 10.1080/09638288.2020.1855263 33280451

[106] Jarman V, Hancock N, Scanlan JN. Maintaining my employment: Learning from people living and working with mental illness. Br J Occup Ther. 2016;79(11):660–668.

[107] Pourhabib A, Sabzi Z, Yazdi K, Fotokian Z. Facilitators and barriers to return to work in patients after heart surgery. J Educ Health Promot. 2022;11(1):310. 36439004 10.4103/jehp.jehp_70_22 PMC9683457

[108] Sarfo M-C, van Asselt KM, Frings-Dresen MH, de Jong F, van Dijk N, de Boer AG. Views of breast cancer survivors on work participation guidance by general practitioners: a qualitative study. BMC Primary Care. 2022;23(1):152. 35715735 10.1186/s12875-022-01768-x PMC9205136

[109] Soeker MS, Wegner L, Pretorius B. I’m going back to work: back injured clients’ perceptions and experiences of their worker roles. Work. 2008;30(2):161–170. 18413932

[110] Andersson C, Jakobsson A, Priebe G, Elf M, Fornazar R, Hensing G. Capability to make well-founded decisions: an interview study of people with experience of sickness absence who have common mental disorders. BMC Public Health. 2022;22(1):1189. 35701748 10.1186/s12889-022-13556-4 PMC9199237

[111] Shaw L, Bondy K, Dodman J. Client insights on knowledge use and access in return to work. Can J Occup Ther. 2009;76(5):359–367.

[112] Müssener U, Ståhl C, Söderberg E. Does the quality of encounters affect return to work? Lay people describe their experiences of meeting various professionals during their rehabilitation process. Work. 2015;52(2):447–455. 26409366 10.3233/WOR-152121

[113] Bratun U, Švajger A, Domajnko B, Kavčič M, Asaba E. Return to work among workers recovering from severe COVID-19 in Slovenia: a focus group study. Disabil Rehabil. 2022;45(23):3883–3892. 10.1080/09638288.2022.2142680. 36346003 10.1080/09638288.2022.2142680

[114] Poulsen AG, Rolving N, Hubeishy MH, Ørtenblad L. Navigating between stakeholders in return-to-work processes: a qualitative study exploring experiences of workers on sick leave due to back pain. Work. 2023;75(4):1277–1287. 10.3233/WOR-220309. 36744359 10.3233/WOR-220309

[115] Aamland A, Werner EL, Malterud K. Sickness absence, marginality, and medically unexplained physical symptoms: a focus-group study of patients’ experiences. Scand J Prim Health Care. 2013;31(2):95–100. 23659708 10.3109/02813432.2013.788274 PMC3656402

[116] Henry AD, Lucca AM. Facilitators and barriers to employment: the perspectives of people with psychiatric disabilities and employment service providers. Work. 2004;22(3):169–182. 15156083

[117] McKay G, Knott V, Delfabbro P. Return to work and cancer: the Australian experience. J Occup Rehabil. 2013;23(1):93–105. 22996341 10.1007/s10926-012-9386-9

[118] Shaw WS, Robertson MM, Pransky G, McLellan RK. Employee perspectives on the role of supervisors to prevent workplace disability after injuries. J Occup Rehabil. 2003;13(3):129–142. 12966688 10.1023/a:1024997000505

[119] Donker-Cools BH, Schouten MJ, Wind H, Frings-Dresen MH. Return to work following acquired brain injury: the views of patients and employers. Disabil Rehabil. 2018;40(2):185–191. 27830952 10.1080/09638288.2016.1250118

[120] Svensson T, Karlsson A, Alexanderson K, Nordqvist C. Shame-inducing encounters. Negative emotional aspects of sickness-absentees’ interactions with rehabilitation professionals. J Occup Rehabil. 2003;13(3):183–195. 12966692 10.1023/a:1024905302323

[121] Sjöström R, Melin-Johansson C, Asplund R, Alricsson M. Barriers to and possibilities of returning to work after a multidisciplinary rehabilitation programme: a qualitative interview study. Work. 2011;39(3):243–250. 21709360 10.3233/WOR-2011-1172

[122] Andersson C, Mårtensson L. Womenʼs experiences of being in the sick leave process. Scand J Occup Ther. 2021;28(6):488–497. 32297821 10.1080/11038128.2020.1750692

[123] Duijts SF, van Egmond MP, Gits M, van der Beek AJ, Bleiker EM. Cancer survivors’ perspectives and experiences regarding behavioral determinants of return to work and continuation of work. Disabil Rehabil. 2017;39(21):2164–2172. 27596990 10.1080/09638288.2016.1219924

[124] Abma FI, Bültmann U, Varekamp I, van der Klink JJ. Workers with health problems: three perspectives on functioning at work. Disabil Rehabil. 2013;35(1):20–26. 22620284 10.3109/09638288.2012.687027

[125] Bartys S, Edmondson A, Burton K, Parker C, Martin R. Work conversations in healthcare: how, where, when and by whom: a review to understand conversations about work in healthcare and identify opportunities to make work conversations a part of everyday health interactions. Public Health England publications; 2019.

[126] Sanerma P, Miettinen S, Paavilainen E, Åstedt-Kurki P. A client-centered approach in home care for older persons—an integrative review. Scand J Prim Health Care. 2020;38(4):369–380. 33201752 10.1080/02813432.2020.1841517 PMC7781976

[127] Holmlund L, Guidetti S, Eriksson G, Asaba E. Return-to-work: exploring professionals’ experiences of support for persons with spinal cord injury. Scand J Occup Ther. 2021;28(7):571–581. 32755475 10.1080/11038128.2020.1795245

[128] Hugenholtz NI, Schaafsma FG, Nieuwenhuijsen K, van Dijk FJ. Effect of an EBM course in combination with case method learning sessions: an RCT on professional performance, job satisfaction, and self-efficacy of occupational physicians. Int Arch Occup Environ Health. 2008;82(1):107–115. 18386046 10.1007/s00420-008-0315-3 PMC2467503

[129] Chou L, Ellis L, Papandony M, Seneviwickrama KMD, Cicuttini FM, Sullivan K, et al. Patients’ perceived needs of osteoarthritis health information: a systematic scoping review. PLoS ONE. 2018;13(4): e0195489. 29659609 10.1371/journal.pone.0195489 PMC5901923

[130] de Kock CA, Lucassen PL, Spinnewijn L, Knottnerus JA, Buijs PC, Steenbeek R, et al. How do Dutch GPs address work-related problems? A focus group study. Eur J General Pract. 2016;22(3):169–175. 10.1080/13814788.2016.1177507 27248862

[131] Papandony MC, Chou L, Seneviwickrama M, Cicuttini FM, Lasserre K, Teichtahl A, et al. Patients’ perceived health service needs for osteoarthritis (OA) care: a scoping systematic review. Osteoarthr Cartil. 2017;25(7):1010–1025. 10.1016/j.joca.2017.02.799 28232144

[132] Hoving JL, van Zwieten MC, van der Meer M, Sluiter JK, Frings-Dresen MH. Work participation and arthritis: a systematic overview of challenges, adaptations and opportunities for interventions. Rheumatology. 2013;52(7):1254–1264. 23472042 10.1093/rheumatology/ket111

[133] Truglio-Londrigan M, Slyer JT, Singleton JK, Worral P. A qualitative systematic review of internal and external influences on shared decision-making in all health care settings. JBI Evid Synth. 2012;10(58):4633–4646. 10.11124/jbisrir-2012-432 27820528

[134] Gibbons C, Porter I, Gonçalves-Bradley DC, Stoilov S, Ricci-Cabello I, Tsangaris E, et al. Routine provision of feedback from patient-reported outcome measurements to healthcare providers and patients in clinical practice. Cochrane Database Syst Rev. 2021;10:CD011589. 10.1002/14651858.CD011589.pub2/full. 34637526 10.1002/14651858.CD011589.pub2 PMC8509115

[135] Malm U, Ivarsson B, Allebeck P, Falloon I. Integrated care in schizophrenia: a 2-year randomized controlled study of two community-based treatment programs. Acta Psychiatr Scand. 2003;107(6):415–423. 12752017 10.1034/j.1600-0447.2003.00085.x

[136] Nieuwenhuijsen K, Hulshof CT, Sluiter JK. The influence of risk labeling on risk perception and willingness to seek help in an experimental simulation of preventive medical examinations. Patient Educ Couns. 2018;101(7):1291–1297. 29471980 10.1016/j.pec.2018.02.011

[137] Faber E, Burdorf A, van Staa AL, Miedema HS, Verhaar JA. Qualitative evaluation of a form for standardized information exchange between orthopedic surgeons and occupational physicians. BMC Health Serv Res. 2006;6(1):1–8. 17081281 10.1186/1472-6963-6-144 PMC1635707

[138] Schwarze M, Spallek M, Korallus C, Manecke I-A, Teumer F, Wrbitzky R, et al. Advantages of the JobReha discharge letter: an instrument for improving the communication interface in occupational rehabilitation. Int Arch Occup Environ Health. 2013;86(6):699–708. 22890776 10.1007/s00420-012-0805-1

[139] Serenko N, Fan L. Patients’ perceptions of privacy and their outcomes in healthcare. Int J Behav Healthc Res. 2013;4(2):101–122.

[140] Chou L, Ranger TA, Peiris W, Cicuttini FM, Urquhart DM, Sullivan K, et al. Patients’ perceived needs of health care providers for low back pain management: a systematic scoping review. Spine J. 2018;18(4):691–711. 29373836 10.1016/j.spinee.2018.01.006

[141] Vooijs M, van Kesteren N, Hazelzet AM, Otten W. Shared decision making from reintegration professionals’ perspectives to support return to work: a qualitative study. BMC Public Health. 2021;21(1):1–10. 33563251 10.1186/s12889-021-10365-z PMC7874602

[142] Silverman J, Kurtz S, Draper J. Skills for communicating with patients. CRC press; 2016.

[143] Carr DD. Motivational interviewing supports patient centered-care and communication. J N Y State Nurses Assoc. 2017;45(1):39–43.

[144] Stern C, Kleijnen J. Language bias in systematic reviews: you only get out what you put in LWW. JBI Evid Synth. 2020;18(9):1818–1819. 10.11124/JBIES-20-00361. 32925418 10.11124/JBIES-20-00361

[145] Porter ME. Value-based health care delivery. Ann Surg. 2008;248(4):503–509. 18936561 10.1097/SLA.0b013e31818a43af

[146] Vogel BA, Bengel J, Helmes AW. Information and decision making: patients’ needs and experiences in the course of breast cancer treatment. Patient Educ Couns. 2008;71(1):79–85. 18191933 10.1016/j.pec.2007.11.023

[147] Sarfo M-C, Bertels L, Frings-Dresen MH, de Jong F, Blankenstein AH, van Asselt KM, et al. The role of general practitioners in the work guidance of cancer patients: views of general practitioners and occupational physicians. J Cancer Surv. 2022;17:416–424. 10.1007/s11764-022-01211-1. 10.1007/s11764-022-01211-1 PMC9038171 35469363

