# Supplementary material for: Patients’ Needs Regarding Work-Focused Healthcare: A Qualitative Evidence Synthesis
Source: J Occup Rehabil. 2024 Jul 25;35(3):450–68. doi: 10.1007/s10926-024-10225-8 (PMC12361345; doi:10.1007/s10926-024-10225-8)
Supplement: Supplementary file 1 — Supplementary file1 (DOCX 353 KB) [file 10926_2024_10225_MOESM1_ESM.docx]

Supplementary appendix

Clients’ needs regarding work-focused healthcare: a qualitative evidence synthesis

Content

Appendix Table 1 – Search strategy Ovid MEDLINE….…………………………………..2

Appendix Table 2 – Study characteristics and data extraction…………………..….3

Appendix Table 3 – Results Critical Appraisal Skills Programme (CASP) qualitative checklist……………………………………….…………………………………………….13

Appendix Table 4 – Evidence profile table……………………………………………...……15

References……………………………………………………………………………………………………21

Supplemental material: Appendix Table 1 – Search strategy Ovid MEDLINE

| **Category** | **#** | **Search** |
| --- | --- | --- |
| Qualitative research | 1 | exp *Qualitative Research/ or interview?.hw. or *Grounded Theory/ |
|  | 2 | (interview* or qualitative or grounded theory or focus group? or group session? or hermeneutic*).mp. |
|  | 3 | (interpretative or phenomenological).ab,kw,ti. |
|  | 4 | or/1-3 |
| Patient perspective [1] | 5 | patient satisfaction/ |
|  | 6 | ((individual or patient? or people or worker?) adj15 (view? or opinion? or satisf*)).ab,kw,ti. |
|  | 7 | ((survivor? or person? or employee? or patient? or absentee? or worker? or people or man or men or wom?n or individual? or participant? or adult?) adj3 (perspective? or barrier? or facilitator? or wants or needs or expect* or percept* or perceive? or views or thoughts or factor? or encounter* or disclos* or discourse or describe? or variable? or experience?)).ab,kw,ti. |
|  | 8 | or/5-7 [perspective] |
| Challenges in work participation | 9 | employment.hw. or *Absenteeism/ or *vocational rehabilitation/ or *medical Leave/ or *Vocational Guidance/ or *Presenteeism/ or *Occupational Health Service/ or exp *Occupational Health/ or *Occupational Therapy/ or *Occupational Medicine/ or *Work Capacity/ |
|  | 10 | (sick leave or work ability or workability or ability to work or work disability or work participation or work functioning or absenteeism or "return* to work" or work return or "stay* at work" or work rehabilitation or unemploy* or work reintegration or "back to work" or sickness absence or presenteeism).ab,kw,ti. |
|  | 11 | or/9-10 [work related] |
| Patient perspective [2] | 12 | ((survivor? or person? or employee? or patient? or absentee? or worker? or people or man or men or wom?n or individual? or participant? or adult?) adj2 interview*).ab,kw,ti. |
|  | 13 | ((factor? or barrier? or facilitator?) and (work functioning or rtw or "return* to work")).ab,kw,ti. |
|  | 14 | and/4,8,11 |
|  | 15 | and/4,11-12 |
|  | 16 | and/4,11,13 |
|  | 17 | or/14-16 |

Supplemental material: Appendix Table 2 – Study characteristics and data extraction

| Ref. | Author (year),  Country | Study aim | Qualitative data collection method | Amount, age, disease & work status of included clients. | Work-focused healthcare  professionals mentioned |
| --- | --- | --- | --- | --- | --- |
| (1) | Aamland et al. (2013), Norway | To explore factors which may influence further marginalization among patients with MUPS on long-term sickness absence. | Focus groups | N = 12, 24-59 years, medically unexplained physical symptoms.  Employed, on sick leave. | Doctors, Norwegian Labour and Welfare Administration officers |
| (2) | Abma et al. (2013), Netherlands | To explore (i) why it is that one worker with a health problem is able to stay at work while the other is not, (ii) to identify signals for decreased functioning at work, and (iii) to explore if and how this can be measured. | Focus groups | N = 7, 47 ± 14.4 years, range of medical conditions.  Employed, coping with problems while staying at work. | Occupational physician |
| (3) | Aguiar-Fernandez et al. (2021), Spain | To analyze experience of returning to work of women who had overcome breast cancer, whereby identifying: 1) the extent to which physical/psychological consequences affected them, 2) personal motivations to return to work, 3) different options/pathways chosen to go back to work, and 4) difficulties encountered. | Focus groups | N = 19, 30-55 years, breast cancer.  Combination of employment status. | Medical, psychological and social services |
| (4) | Amir et al. (2008),  UK | To explore the context of the UK’s social welfare system, have returned to the world of work. | Individual interviews | N = 41, 26-55 years, cancer.  Employed, returned to work after sick leave. | Cancer care team, general practitioner, occupational health practitioner |
| (5) | Andersson et al. (2022), Sweden | To investigate the prerequisites for support, knowledge, and information related to decision making experienced by people on sickness absence due to common mental disorders (CMDs). | Individual interviews | N = 15, 20-55 years, common mental disorders.  Combination of employment status. | Physicians, healthcare system, Social insurance agency |
| (6) | Andersson & Mårtensson (2021), Sweden | To explore the experiences of being on sick leave among a group of women. | Individual interviews | N = 13, 48 (31–63) years, range of medical conditions.  Combination of employment status. | Health professional |
| (7) | Audhoe et al. (2018), Netherlands | To evaluate the barriers to and solutions for return to work (RTW) from the perspective of unemployed workers who were sick-listed due to mental health problems. | Individual interviews | N = 25, 43 (22-59) years, mental health problems.  Unemployed, on sick leave. | Social security agency, vocational rehabilitation agencies |
| (8) | Bae & Cho (2021),  Korea | To understand the unmet needs of cancer patients and identify the necessary factors to develop a vocational intervention program based on cancer patients’ work-related experience after cancer diagnosis. | Individual interviews | N = 50, 45.72 ± 7.51 years, cancer.  Combination of employment status. | Medical staff, the doctor |
| (9) | Bardgett et al. (2016),  UK | To gain an insight into the factors influencing return to work following knee replacement from the patients' perspective. | Individual interviews | N = 10, 40-59 years, knee replacement.  Employed, returned to work after sick leave. | Healthcare professionals, general practitioner, nurse practitioner, clinician, occupational health worker |
| (10) | Beaulieu (2016),  UK | To explore the experiences of return to paid work after a brain injury. | Individual interviews | N = 16, median 47 years, Moderate or severe traumatic or acquired brain injury.  Employed, returned to work after sick leave. | Social work, occupational therapy, headway UK, occupational health. |
| (11) | Beerda et al. (2021), The Netherlands | To qualitatively explore experiences and perspectives of patients with advanced cancer regarding work resumption and work retention. | Individual interviews | N = 11, 52 (41-64) years, advanced cancer.  Combination of employment status. | Occupational physicians, clinicians |
| (12) | Bennink et al. (2021), The Netherlands | To explore perspectives and experiences of patients with multiple myeloma and (health care) experts regarding RTW and participation at work. | Individual interviews | N = 9, 54.2 ± 4.2 years, multiple myeloma.  Combination of employment status. | Occupational physicians, insurance physicians, healthcare professionals in the hospital |
| (13) | Berger et al. (2020), Canada | To explore the needs of cancer survivors when returning to or staying in the workforce, by examining cancer survivors’ perspectives on supports needed when returning to or staying in the workforce. | Both, individual interviews and focus groups. | N = 15, 25-64 years, cancer.  Combination of employment status. | Insurance companies, healthcare professionals, family doctor |
| (14) | Blokzijl, et al. (2021), Netherlands | To explore the barriers that obstruct return to work after coronary artery bypass grafting. | Individual interviews | N = 10, 49-62 years, Coronary artery bypass grafting.  Employed, returned to work after sick leave. | Healthcare professionals, surgeons, cardiologist, occupational physician, cardiac rehabilitation, general practitioner |
| (15) | Bosma et al. (2020), Netherlands | To explore the lived experiences of workers with a chronic condition and identify existing barriers, facilitators and possible support needs for staying at work. | Focus groups | N = 30, 46.6 (23-73) years, One or more chronic conditions.  Employed, coping with problems while staying at work | Occupational physicians (company doctor), healthcare professionals and medical specialists, Dutch social security institute, (rehabilitation doctor) |
| (16) | Brakenridge et al. (2021), Australia | To explore work outcomes, vocational services, barriers and facilitators for returning to work in individuals with acquired brain injury (ABI) in Queensland, Australia and to identify areas for improvement. | Individual interviews | N = 10 22-62 years, acquired brain injury.  Combination of employment status. | Vocational rehabilitation support, occupational therapist, physiotherapists, supportive rehabilitation officer/case manager |
| (17) | Bratun et al. (2022), Slovenia | To explore expectations and experiences regarding RTW during the rehabilitation process among a group of workers recovering from severe COVID-19. | Focus groups | N = 18, 51.2 (39-65) years, severe COVID-19.  Combination of employment status. | RTW system in place, various experts playing important parts in the process of RTW and rehabilitation |
| (18) | Bridger et al. (2021),  UK | Identify outcomes of vocational rehabilitation interventions that are important to survivors. | Both, individual interviews and focus groups. | N = 17, 44 (27-68) years, trauma survivors.  Combination of employment status. | Psychological support |
| (19) | Coole et al. (2010a),  UK | To explore the experiences of employed people with back pain and their perceptions of how GPs and other clinicians have addressed their work difficulties. | Individual interviews | N = 25, 44.7 (22-58) years, low back pain.  Combination of employment status. | General practitioner, other clinicians (as chiropractor, physiotherapist), occupational health professional |
| (20) | Coole et al. (2010b),  UK | To explore employed patients’ experiences and perceptions of work, prior to attending a rehabilitation programme. | Individual interviews | N = 25, 44.7 (22-58) years, low back pain.  Combination of employment status. | Occupational physician, staff nurse, general practitioner, |
| (21) | Corbiere et al. (2022), Canada | To (1) document meaning of experience of recognition in the RTW process of work after a sick leave due to a CMD, and (2) investigate phenomenon of work recognition for workers in the process of RTW after a sick leave due to a CMD, by evaluating the presence or absence of marks of recognition from salient RTW stakeholders stemming from different systems. | Individual interviews | N = 20, 39 (26-53 years), common mental disorders.  Employed, returned to work after sick leave | General practitioner, mental health professionals, insurers |
| (22) | Decuman et al. (2015), Belgium | To describe, from the patient’s point of view, the factors influencing the occupational trajectory of pa- tients with systemic sclerosis (SSc). | Individual interviews | N = 14, 49 (35-71) years, Systemic Sclerosis.  Combination of employment status. | Medical professional, physician, medical adviser |
| (23) | Dewa et al. (2018),  Canada | To explore the return- to-work (RTW) experience of head and neck cancer (HNC) survivors. | Individual interviews | N = 9, 23-66 years, head and neck cancer survivors.  Employed, returned to work after sick leave. | Treating physician |
| (24) | Donker-Cools et al. (2018), Netherlands | To investigate which factors are experienced as facilitators of or barriers to return to work (RTW), or as solutions to RTW-problems, by patients with acquired brain injury (ABI) and by employers. | Individual interviews | N = 10, 47 (34-63) years, non-progressive acquired brain injury.  Employed, returned to work after sick leave. | Treating physicians, occupational physicians, insurance physicians |
| (25) | Dorland et al. (2016), Netherlands | Identify barriers and facilitators of work functioning among CSs. | Focus groups | N = 22, 47.2 ±7.4 years, cancer survivors.  Employed, returned to work after sick leave. | Occupational physicians |
| (26) | Duijts et al. (2017), Netherlands | To explore cancer survivors’ perspectives and experiences regarding behavioral determinants of RTW and continuation of work. | Individual interviews | N = 28, 52 (28-62) years, cancer survivors.  Combination of employment status. | Occupational physician |
| (27) | Frazier et al. (2009),  USA | To identify themes from the employment experiences of cancer survivors that suggest ways in which frontline oncology physicians and nurses could help patients with job issues in the months after diagnosis. | Focus groups | N = 73, age not specified, cancer survivors.  Employed, coping with problems while staying at work. | Physician, oncology nurses |
| (28) | Gard et al. (2019),  Sweden | To explore stroke survivors’ experiences of healthcare-related facilitators and barriers concerning return to work after stroke. | Focus groups | N = 20, median age 52, stroke.  Employed, returned to work after sick leave. | Rehabilitation actors, interdisciplinary rehabilitation team (psychotherapy, mindfulness, occupational therapist, psychologist, cognitive behavior therapy), healthcare professionals, social insurance office, outpatient stroke rehabilitation, rehabilitate |
| (29) | Gilworth et al. (2008),  UK | To explore the work-related expectations and experiences of workers who had sustained mild to moderate brain injury. | Individual interviews | N = 33, 37(19-55) years, mild to moderate traumatic brain injury.  Combination of employment status. | Not known |
| (30) | Gilworth et al. (2009),  UK | Investigating the expectations and experiences of stroke survivors in relationship to return to work. | Individual interviews | N = 13, 50.8 (24-64) years, Cerebrovascular disease.  Combination of employment status. | Hospital staff, health care team, doctor, general practitioner, consultant |
| (31) | Graff et al. (2021), Denmark | Targeting experiences of how different actors facilitated or acted as a barrier in the RTW process, and encompassed the mTBI trajectory from the acute phase to the post-acute phase. | Individual interviews | N = 22, age not specified, mild traumatic brain injury, 2-5 years post injury.  Combination of employment status. | General practitioner, social worker, municipality (refer to: physiotherapist, personal trainer, stress management therapy, and neuropsychological assessments), coordinator |
| (32) | Hartke et al. (2011),  USA | Describe the facilitators and barriers that stroke survivors encounter in their efforts to RTW. | Individual interviews | N = 12, 51 (31-67) years, stroke.  Combination of employment status. | Health care professionals, doctors and therapists, vocational counselor, vocational advocates |
| (33) | Hellman et al. (2016), Sweden | To explore and describe important aspects expressed by Swedish professionals in the RTW process for persons post stroke and to contrast the professionals’ aspects exploring different perspectives that may influence optimal RTW. | Focus groups | N = 20, age not specified, stroke.  Employed, on sick leave. | Agencies |
| (34) | Henry & Lucca (2004),  USA | Examine the perspectives of people with psychiatric disabilities and employment service providers regarding factors that most directly help or hinder consumer efforts to obtain and maintain employment. | Focus groups | N = 44, 41.1 ±8.37 (22-59) years, serious mental illness.  Combination of employment status. | Service providers, DSS, social security system, social security administration workers |
| (35) | Hjärtström et al. (2018), Sweden | To explore the relationship between work and chronic health conditions in a group of employees aged 50–64 years with a focus on factors that enable them to continue to work. | Individual interviews | N = 10, 59.5 (51-63) years, one or more chronic conditions.  Employed, returned to work after sick leave. | Company's occupational healthcare providers, company's healthcare team, healthcare agencies, such as primary care, specialists, and the occupational healthcare providers, social insurance fund |
| (36) | Holmlund et al. (2018), Sweden | To explore experiences of return to work in the context of everyday life among adults 7–11 years after spinal cord injury (SCI). | Individual interviews | N = 8, 34 (27-41) years, traumatic spinal cord injury.  Combination of employment status. | Administrator at the social insurance office |
| (37) | Holmlund et al. (2020), Sweden | To identify facilitators of and barriers to the coordination of return-to-work between the primary care services, the employee, and the employers. | Individual interviews | N = 9, 25-61 years, Common mental disorders .  Employed, on sick leave. | Primary care services, professionals at the Primary care center |
| (38) | Hooson et al. (2013),  UK | To explore the experience of return to work rehabilitation with individuals who sustained TBI. | Individual interviews | N = 10, 43.1 ±13.8 (23-62) years, Traumatic brain injury.  Combination of employment status. | Clinical team, occupational health department, company doctor, occupational therapist, clinicians (occupational therapy and psychology and speech and language therapist and physio) |
| (39) | Hubertsson et al. (2011), Sweden | Study how patients with personal experience of sickness absence due to musculoskeletal disorders perceived their contact with these actors and what they considered as obstructing or facilitating factors for recovery and return to work | Individual interviews | N = 15, 33-63 years, Musculoskeletal disorders.  Combination of employment status. | Social Insurance Agency, social insurance officers, health care system, social workers, treating physician, psychological support, 'spider in a web' |
| (40) | Jain et al. (2019),  India | To explore the extent to which rheumatoid arthritis (RA) impacts work productivity in patients living with RA in India. | Individual interviews | N = 20, 40 (35.7–46.5) years, Rheumatoid arthritis.  Employed, coping with problems while staying at work. | Department (health care), doctors, clinicians, government, physiotherapy, occupational rehabilitation |
| (41) | Jansson & Björklund (2007), Sweden | To explore from an environmental perspective the experiences of returning to work of former unemployed sickness absentees. | Focus groups | N = 16, age not specified, range of handicaps.  Employed, returned to work after sick leave. | Professionals, employment office |
| (42) | Jarman et al. (2016), Australia | Gain understanding of the experiences of people living with mental illness who have managed to maintain their employment, and specifically, the strategies they actively choose and use to stay in work. | Individual interviews | N = 10, 41.5 ±12 (23-56) years, Mental illness and psychiatric diagnosis.  Employed, returned to work after sick leave | Employment specialist, psychiatrist, mental health clinician |
| (43) | Joosen et al. (2022), The Netherlands | To investigate 1) what workers with common mental disorders (CMDs) perceive as causes for their sickness absence, 2) what these workers perceives as barriers and facilitators for their RTW, and 3) how perceptions differ between workers with short (<3 months), medium (3-6 months), and long term (> 6 months) sickness absence. | Individual interviews | N = 34, 49 (29-62) years, common mental disorders.  Combination of employment status | Occupational physicians, occupational health professionals |
| (44) | Juurlink et al. (2019), Netherlands | To explore barriers and facilitators in gaining and maintaining employment in borderline personality disorder. | Individual interviews | N = 15, 39 (23–58) years, borderline personality disorder.  Combination of employment status. | Mental health care system, vocational rehabilitation, social security agency, insurance physicians |
| (45) | Karcz et al. (2022), Switzerland | To identify factors influencing sustainable employment experienced by people with spinal cord injury or acquired brain injury. | Both, individual interviews and focus groups | N = 51, age not specified, spinal cord injury or acquired brain injury.  Combination of employment status. | Case managers, cantonal IV office, family physicians, psychiatrists, support systems provided by patient organizations, health accident and disability insurances, insurance or vocational integration specialist, qualified support person who is knowledgeable about the procedures of the health and social security system |
| (46) | Kennedy et al. (2007),  UK | To explore the factors that influence decisions about return to work either during or after cancer treatment and to identify the important aspects of returning to work. | Both, individual interviews and focus groups. | N = 29, mean age 52.6, cancer.  Combination of employment status. | Healthcare professionals, doctor or specialist, secretary |
| (47) | Klaver et al. (2020), Netherlands | Generate in-depth information regarding cancer-related cognitive problems in working cancer survivors, strategies used to cope with cancer-related cognitive problems at work, and needs of cancer survivors and professionals regarding cancer-related cognitive problems at work. | Focus groups | N = 23, 49.5 ± 11.4 (31-70) years, cancer.  Employed, returned to work after sick leave. | Psychologist, coach, occupational physician, (occupational) health care professionals, occupational therapist |
| (48) | Kluit et al. (2022), The Netherlands | To develop a better understanding of the current practices, needs, and expectations surrounding discussing work with a medical specialist from a patient’s perspective. | Focus groups. | N = 33, 30-67 years, diverse medical histories.  Combination of employment status. | Medical specialist, healthcare professionals in the hospital, occupational physician, rehabilitation physician, private clinics |
| (49) | Knott et al. (2014), Australia | To explore barriers to return to work and preferences for intervention and support for cancer patients treated with curative intent from the perspectives of cancer survivors and oncology health professionals. | Both, individual interviews and focus groups. | N = 17, 52.3 ± 10.2 years, cancer.  Employment status not known. | Occupational health physicians, contact persons (advocate from the hospital) |
| (50) | Libeson et al. (2020), Australia | Understand the return to work (RTW) experience of individuals with TBI who received comprehensive vocational rehabilitation, and to identify facilitating and limiting factors in the RTW process. | Individual interviews | N = 15, mean age 47.3, moderate of severe traumatic brain injury.  Combination of employment status. | (RTW programme) TBI-specialized occupational therapist, neuropsychologist |
| (51) | Lindahl et al. (2013), Denmark | Investigate what constitutes good quality in rehabilitation after a person has sustained a fracture at working age, from both patients’ and therapists’ perspectives. | Individual interviews | N = 7, 51 (32-60) years, bone fracture.  Combination of employment status. | Therapists |
| (52) | Lock et al. (2005),  UK | To explore stroke survivors’ own perspectives about what helps and hinders paid or voluntary work after stroke. | Focus groups | N = 37, age not specified, stroke.  Employment status not known. | Rehabilitation professionals, occupational therapists, vocational rehabilitation |
| (53) | Lork & Holmgren (2018),  Sweden | To explore how individuals on sick leave experience their return to work self-efficacy. | Individual interviews | N = 9, 45 (30-65) years, physical and mental illness, road accidents and chronic fatigue syndrome.  Employed, on sick leave. | Social insurance system, various authorities, healthcare system |
| (54) | Lysaght & Larmour-Trode (2008), Canada | To explore workplace disability support from worker and supervisory perspectives and to identify salient features for work re-entry. | Individual interviews | N = 18, 47.7 (24-61) years, more than one workplace injury during work history.  Employed, returned to work after sick leave. | Physician, occupational health nurse, physiotherapist, occupational health coordinator |
| (55) | MacLennan et al. (2021), UK | To explore the lived experience of work after treatment for breast cancer in a group of professional working women within the UK. | Individual interviews | N = 15, 51.7 (39-59) years, breast cancer.  Combination of employment status. | Healthcare providers, primary and secondary providers, occupational health |
| (56) | Madsen et al. (2023), Denmark | To explore challenges at work and identify the need for professional support among Danish people with inflammatory arthritis. | Individual interviews | N = 15, (36-68) years, inflammatory arthritis  Combination of employment status | Coordinator from outpatient rheumatology, occupational therapist, physiotherapist, nurse, social worker at municipal job center |
| (57) | Maillette et al. (2017), Canada | Document workers’ representations or understanding of work disability after total knee arthroplasty. | Individual interviews | N = 8, 55 (42-62) years, knee arthroplasty.  Combination of employment status. | Physical therapist, orthopedic surgeon, insurers |
| (58) | Mansfield et al. (2014), Canada | To explore the experiences of individuals who had suffered an electrical injury at work and had subsequently returned to work. | Individual interviews | N = 13, age not specified, electrical injury.  Employment status not known. | Rehabilitation service provided by specialized electrical-injury clinics, healthcare providers, workers' compensation representatives, family physician |
| (59) | Mårtensson & Hensing (2012), Sweden | To explore and describe women’s experiences of factors that contribute to their ability to make informed decisions about the process of rehabilitation and return to work. | Focus groups | N = 19, 51 (26-63) years, range of medical conditions.  Combination of employment status. | Certain persons working at healthcare centers, healthcare professionals, sick-listing physician or social insurance administrator, social insurance office, coach |
| (60) | McKay et al. (2013), Australia | Gain an understanding of the return to work process and factors that affect the experience. | Both, individual interviews and focus groups. | N = 15, 27–69 years, cancer.  Combination of employment status. | Mediator such as a doctor or psychologist |
| (61) | McRae et al. (2016), Australia | Investigate the vocational rehabilitation experiences of clients, up to 14 years post brain injury. | Individual interviews | N = 29, 35.8 ±13.0 (19–66) years, traumatic brain injury up to 14 years post trauma.  Combination of employment status. | staff with specialist knowledge about acquired brain injury, psychological counselling, VR providers |
| (62) | Medin et al. (2006), Sweden | Describe the experience of return to work after stroke from the patient’s perspective. | Individual interviews | N = 6, age not specified, stroke.  Employment status not known. | Rehabilitation professionals, social insurance officers, occupational therapist |
| (63) | Miller et al. (2023), UK | To investigate head and neck cancer survivors’ experiences following a return to work, and establish priorities for practice, and situate knowledge within context. | Individual interviews | N = 13, 54 (39-63) years, head and neck cancer.  Employed, returned to work after sick leave. | Healthcare professionals. |
| (64) | Müssener et al. (2007), Sweden | Identify and analyze sick-listed persons’ experiences of positive encounters with professionals within social insurance and healthcare. | Individual interviews | N = 11, 28-59 years, range of medical conditions.  Combination of employment status. | Professionals both within healthcare and the social insurance system, contact at the social insurance office, doctors, healthcare professional, social insurance officers, doctor, physicians |
| (65) | Müssener et al. (2015), Sweden | Gain a deeper understanding of how individuals with experiences of being on sick leave perceive their encounters with professionals, and how such encounters affected their ability to return to work, as well as their attitudes towards | Individual interviews | N = 20, 33-59 years, range of medical conditions.  Combination of employment status. | Professionals, physicians, physiotherapist, rehabilitation |
| (66) | Netto et al. (2016), Austalia | Generate a rich description of the meaning of employment for people with mental illness and identify the facilitators and barriers they experience in gaining and sustaining employment. | Both, individual interviews and focus groups. | N = 9, 33-55 years, mental illness.  Combination of employment status. | Employment consultants, service providers, mental health care service providers, staff from vocational activities |
| (67) | Newington et al. (2019), UK | To explore the return to work experiences of patients who had recently undergone carpal tunnel release surgery. | Individual interviews | N = 14, 27-68 years, Carpal tunnel release surgery.  Employed, returned to work after sick leave. | Surgeon, clinician |
| (68) | Nilsson et al. (2011), Sweden | Gain knowledge about women’s experiences of encounters regarding return to work after breast cancer surgery. | Focus groups | N = 23, 53 (37-62) years, breast cancer.  Combination of employment status. | Social insurance officers, healthcare personnel as physicians, nurses and social workers |
| (69) | Noordik et al. (2011), Netherlands | Describe the barriers to a full return to work, solutions, communicating to the working environment and the aim of a full return to work. | Individual interviews | N = 15, 38 (25-58) years, stress-related disorders.  Employed, on sick leave. | Occupational health staff, psychologist, occupational physician |
| (70) | Nouri et al. (2020),  UK | To investigate patients’ views and experiences of work-related advice provided by clinicians, and how this might be improved. | Individual interviews | N = 45, 59.8 (43-76) years, total hip and knee replacement.  Employed, on sick leave. | Orthopedic teams, staff, clinicians, healthcare professionals, occupational health |
| (71) | Olischläger et al. (2022), The Netherlands | To explore experiences and needs of: (1) patients with a rare cancer regarding return to work and work retention, and (2) healthcare professionals regarding work-related support of patients with a rare cancer, throughout their disease trajectory. | Individual interviews | N = 16, 49 (30-64) years, rare cancer.  Combination of employment status | Healthcare professionals, occupational physicians (specialized in oncology), oncology nurse, representative of a specialized occupational rehabilitation service |
| (72) | Oosting et al. (2023), The Netherlands | To gain insight into patient's experiences, needs, and expectations regarding cooperation between clinical and occupational health care practitioners with a focus on medical specialists and occupational health physicians. | Focus groups | N = 33, (30-67) years, different medical histories.  Combination of employment status | Occupational health physicians, medical specialists, health care practitioners |
| (73) | Öster et al. (2010),  Sweden | To explore burn-injured individuals’ perception of factors seen as facilitators or barriers in the process of returning to work after a severe burn injury. | Individual interviews | N = 39, 39.7 ± 10.3 (19–61) years, burn injury,  combination of employment status. | Acute care, primary health care, rehabilitation, physiotherapy, psychological support, social insurance office, job centers, insurance companies |
| (74) | Österholm et al. (2013), Sweden | To explore the perception of the ability to maintain working by men with arthritis. | Individual interviews | N = 9, 48 (31-62) years, arthritis.  Employment status not known. | The authorities (social insurance office and the Swedish public employment service), health care service, health care professionals |
| (75) | Pahlplatz et al. (2021), The Netherlands | To identify patients’ perspectives regarding beneficial and limiting factors in return to work after total knee arthroplasty. | Focus groups | N = 17, (52-65) years, total knee arthroplasty.  Combination of employment status. | Physical therapists, Employee Insurance Agency, orthopedic surgeon |
| (76) | Pasanen (2021), Finland | To explore encounters between disabled workers and insurers within an earnings-related pension scheme in Finland in order to enhance understanding of the lived experience of positive encounters during the return to work process. | Individual interviews | N = 24, (28-61), different disorders or diseases.  Combination of employment status | Insurers, insurance companies |
| (77) | Poulsen et al. (2022), Denmark | To explore how workers on sick leave due to back pain experience navigating between stakeholders involved in their RTW process, focusing on the job center, the health care system, and the workplace. | Individual interviews | N = 16, (20-65), back pain.  Combination of employment status. | Job center, healthcare system |
| (78) | Pourhabib et al. (2022), Iran | To identify facilitators and barriers to return to work in Iranian patients after heart surgery. | Individual interviews | N = 11, (48-63) years, heart surgery.  Employment status not known. | Physician, cardiac surgeon, insurance companies |
| (79) | Rubenson et al. (2007), Sweden | To explore experiences of returning to work after rehabilitation, from the viewpoint of people with acquired brain injury. | Individual interviews | N = 8, 39 (23-63) years, Acquired brain injury.  Employed, returned to work after sick leave. | Company doctor, occupational therapist, human beings during the rehabilitation process, brain injury team, regional social insurance office |
| (80) | Ryan et al. (2014),  UK | To explore the experiences of individuals returning to work after an episode of sickness absence due to LBP. | Individual interviews | N = 5, age not specified, low back pain.  Employed, returned to work after sick leave. | General practitioner |
| (81) | Sarfo et al. (2022), The Netherlands | To define the role of the general practitioner role in providing work participation and return to work guidance to breast cancer survivors, from the perspective of the breast cancer survivors. | Focus groups | N = 25, 51 ± 7.3 (31-64) years, breast cancer.  Combination of employment status. | General practitioner, occupational health physician |
| (82) | Shaw et al. (2003),  USA | Describe the role of supervisors to prevent workplace disability as part of a needs assessment for a pilot supervisor training program. | Individual interviews | N = 30, age not specified, range of current or past work injuries.  Employed, coping with problems while staying at work. | Medical specialist (doctor, therapist) , adjudicators, compensation board |
| (83) | Shaw et al. (2009),  Canada | Investigate clients’ perspectives on their experiences, needs, and preferences for accessing and using information to make decisions in resuming work and living with disability | Individual interviews | N = 14, 35-56 years, range of chronic disabilities.  Employed, on sick leave. | Professionals from the insurance system and return to work system |
| (84) | Sjöström et al. (2011), Sweden | To explore experiences of sick-listed persons of not returning to work during a six-year period after participation in an extensive multidisciplinary rehabilitation programme. | Individual interviews | N = 10, 48 (29-52) years, Musculoskeletal disorders, neck and back pain.  Employed, on sick leave. | Social insurance office, healthcare professionals |
| (85) | Soeker et al. (2008),  South Afrika | To explore perceptions and experiences of facilitators and barriers that affected individuals who received back rehabilitation and their ability to resume their worker roles. | Focus groups | N = 26, age not specified, back injury.  Employed, returned to work after sick leave. | Medical professionals, physicians, medical practitioners, doctor, insurance systems, treating health professionals (e.g. physiotherapists and physician) |
| (86) | Sturesson et al. (2014), Sweden | To explore how sick-listed patients in Sweden perceive their contact with healthcare professionals in primary healthcare and to analyze what they view as crucial components for returning to work. | Individual interviews | N = 21, 51 (27-63) years, range of musculoskeletal, mental or other disorders.  Combination of employment status. | Healthcare professionals, notional insurance agency |
| (87) | Svensson et al. (2003), Sweden | To explore and describes negative emotions (“shame” in a broad sense) experienced by individuals on sick leave in their interactions with rehabilitation professionals. | Focus groups | N = 18, age not specified, back, neck or shoulder diagnosis.  Employed, on sick leave. | Rehabilitation workers, physician, physiotherapist, doctor, occupational service, social insurance officials |
| (88) | Tamminga et al. (2012), Netherlands | Identify factors experienced as barriers to and facilitators of the return-to-work (RTW) process, which factors were important during initial and post RTW and possible solutions to RTW problems. | Individual interviews | N = 12, 42 ± 7 years, breast cancer.  Combination of employment status. | Occupational physicians, health professionals at the hospital, oncological nurse, treating physicians, reintegration counselors, occupational physician specialized in cancer and work, social worker, psychologist |
| (89) | Urquhart et al. (2022), Canada | To identify strategies policies, and practices that can support cancer survivor’s return to work after cancer treatment, by exploring (1) cancer survivors’ needs related to return to work and how these changed over time, and (2) experiences reintegrating into the workplace and what helps and hinders this process. | Individual interviews | N = 13, age not specified, cancer.  Combination of employment status. | Cancer care team, primary care provider, insurance company |
| (90) | Van der Meer et al. (2011), Netherlands | To investigate the experiences and needs with respect to work participation of employees with rheumatoid arthritis treated with anti-tumor necrosis factor therapy. | Individual interviews | N = 14, 47 ± 2,9 years, Rheumatoid Arthritis on anti-TNF therapy.  Employed, returned to work after sick leave. | Healthcare professionals: rheumatologists, specialized nurses, psychologists, occupational physicians and social workers |
| (91) | Van Egmond et al. (2017), Netherlands | To explore experiences of job loss and to explore barriers and facilitators for return to work in a broad sample of cancer survivors, who have experienced job loss before or during cancer diagnosis and treatment. | Both, individual interviews and focus groups. | N = 17, 51 (31-58) years, cancer survivors.  Combination of employment status. | Insurance physician, occupational physicians, general physician |
| (92) | Vooijs et al. (2017), Netherlands | To explore solutions that people with a chronic disease use to overcome difficulties they experience regarding participating in work, and the support they require to identify or implement these solutions. | Focus groups | N = 19, 50 ± 10.7 (28-62) years, range of chronic medical conditions.  Combination of employment status. | Occupational health professionals, coaches, health professionals |
| (93) | Wallstedt-Paulsson et al. (2007), Sweden | Investigate how clients perceive their work experiences after a one-year follow up in work rehabilitation. | Individual interviews | N = 14, 47-65 years, range of medical conditions.  Employed, on sick leave. | Regional social insurance officer, rehabilitation workers, rehabilitation unit |
| (94) | Watter et al. (2021), Australia | Investigate the experiences of community-living adults with acquired brain injury regarding their vocational rehabilitation and return to work. | Both, individual interviews and focus groups. | N = 8, mean age 45, Acquired brain injury.  Combination of employment status. | Workplace rehab person, rehabilitation, employment services, VR services |
| (95) | Yarker et al. (2010),  UK | To explore the way in which communication and support at work effect cancer survivors on their return to work and during the post-return period. | Individual interviews | N = 26, 31-64 years, cancer.  Employed, returned to work after sick leave | Occupational health departments from work (occupational health nurse), oncology nurse, occupational health, consultant |
| (96) | Zaman et al. (2017), Netherlands | To assess facilitators and barriers that affect the activities of an oncological occupational physician (OOP). | Individual interviews | N = 8, mean age 54, cancer.  Employment status not known. | Oncology occupational physician |
| (97) | Zamanzadeh et al. (2018), Irak | To explore Iranian cancer survivors' experiences of returning to work. | Individual interviews | N = 20, 40.75 ± 11.03 years, cancer.  Employed, returned to work after sick leave. | Health care professionals (doctors and nurses) |

Supplemental material: Appendix Table 3 – Results Critical Appraisal Skills Programme (CASP) qualitative checklist

| Reference |  | | 1. Was there a clear statement of the aims of the research? | 2. Is a qualitative methodology appropriate? | 3. Was the research design appropriate to address the aims of the research? | 4. Was the recruitment strategy appropriate to the aims of the research? | | 5. Was the data collected in a way that addressed the research issue? | | 6. Has the relationship between researcher and participants been adequately considered? | | 7. Have ethical issues been taken into consideration? | 8. Was the data analysis sufficiently rigorous? | 9. Is there a clear statement of findings? | 10. How valuable is the research? | Total |
| --- | --- | --- | --- | --- | --- | --- | --- | --- | --- | --- | --- | --- | --- | --- | --- | --- |
| (1) | | Aamland, 2013 | + | + | + | | ? | | ? | | - | + | + | + | + | 7 |
| (2) | | Abma, 2013 | + | + | + | | - | | + | | - | - | ? | + | + | 6 |
| (3) | | Aguiar-Fernande, 2021 | + | + | + | | + | | + | | ? | + | + | + | + | 9 |
| (4) | | Amir, 2008 | + | + | + | | + | | + | | - | + | + | + | + | 9 |
| (5) | | Andersson, 2020 | + | + | + | | + | | + | | + | + | + | + | + | 10 |
| (6) | | Andersson, 2022 | + | + | ? | | ? | | + | | ? | + | + | + | + | 7 |
| (7) | | Audhoe, 2018 | + | + | ? | | + | | + | | - | + | + | + | + | 8 |
| (8) | | Bae, 2021 | + | + | + | | + | | + | | ? | + | + | + | + | 9 |
| (9) | | Bardgett, 2018 | + | + | ? | | + | | + | | - | + | + | + | + | 8 |
| (10) | | Beaulieu, 2016 | + | + | + | | ? | | + | | ? | + | + | + | + | 8 |
| (11) | | Beerda, 2022 | + | + | ? | | + | | + | | ? | + | + | + | + | 8 |
| (12) | | Bennink, 2021 | + | + | ? | | + | | + | | - | + | + | + | + | 8 |
| (13) | | Berger, 2020 | + | + | + | | ? | | + | | ? | + | + | + | + | 8 |
| (14) | | Blokzijl, 2020 | + | - | + | | ? | | + | | - | + | - | + | + | 6 |
| (15) | | Bosma, 2020 | + | + | + | | + | | + | | - | + | + | + | + | 9 |
| (16) | | Brakenridge, 2021 | + | + | + | | + | | + | | + | + | + | + | + | 10 |
| (17) | | Bratun, 2022 | + | + | + | | + | | + | | - | + | + | + | + | 9 |
| (18) | | Bridger, 2021 | + | + | + | | ? | | + | | ? | + | + | + | + | 8 |
| (19) | | Coole, 2010a | + | + | + | | ? | | + | | - | + | ? | + | + | 7 |
| (20) | | Coole, 2010b | + | + | + | | ? | | + | | - | + | ? | + | + | 7 |
| (21) | | Corbière, 2022 | + | + | ? | | ? | | + | | ? | + | - | + | + | 6 |
| (22) | | Decuman, 2015 | + | + | ? | | ? | | ? | | ? | + | + | - | + | 5 |
| (23) | | Dewa, 2018 | + | + | ? | | ? | | + | | + | + | + | + | + | 8 |
| (24) | | Donker-Cools, 2018 | + | + | ? | | + | | + | | + | + | + | + | + | 9 |
| (25) | | Dorland, 2016 | + | + | ? | | ? | | ? | | ? | + | - | + | + | 5 |
| (26) | | Duijts, 2017 | + | + | ? | | + | | + | | ? | + | + | + | + | 8 |
| (27) | | Frazier, 2009 | + | + | ? | | ? | | ? | | ? | ? | ? | + | + | 4 |
| (28) | | Gard, 2019 | + | + | + | | + | | ? | | + | + | + | + | + | 9 |
| (29) | | Gilworth, 2008 | + | + | + | | + | | - | | ? | + | + | + | + | 8 |
| (30) | | Gilworth, 2009 | + | + | + | | ? | | + | | ? | + | + | + | + | 8 |
| (31) | | Graff, 2020 | + | + | ? | | - | | + | | + | + | + | + | + | 8 |
| (32) | | Hartke, 2011 | + | + | ? | | ? | | + | | ? | + | + | + | + | 7 |
| (33) | | Hellman, 2016 | + | + | + | | - | | ? | | - | + | + | - | - | 5 |
| (34) | | Henry, 2004 | - | + | - | | ? | | + | | - | - | + | + | + | 5 |
| (35) | | Hjartstrom, 2008 | + | + | ? | | ? | | + | | - | + | - | + | - | 5 |
| (36) | | Hulmlund, 2018 | + | + | ? | | + | | + | | - | + | + | + | + | 8 |
| (37) | | Hulmlund, 2020 | + | + | ? | | + | | + | | - | + | + | + | + | 8 |
| (38) | | Hoossen, 2013 | + | + | + | | - | | + | | - | + | ? | + | + | 7 |
| (39) | | Hubertsson, 2011 | + | + | ? | | + | | + | | - | + | + | + | + | 8 |
| (40) | | Jain, 2020 | + | + | ? | | + | | + | | + | + | + | + | + | 9 |
| (41) | | Jansson, 2007 | + | + | + | | ? | | + | | + | + | + | + | + | 9 |
| (42) | | Jarman, 2016 | + | + | ? | | ? | | + | | - | + | + | + | + | 7 |
| (43) | | Joosen, 2022 | + | + | + | | + | | + | | - | + | + | + | + | 9 |
| (44) | | Juurlink, 2019 | + | + | + | | ? | | + | | - | + | + | - | + | 7 |
| (45) | | Karcz, 2022 | + | + | ? | | + | | + | | - | + | + | + | + | 8 |
| (46) | | Kennedy, 2007 | + | + | - | | ? | | + | | - | - | + | - | + | 5 |
| (47) | | Klaver, 2020 | + | + | ? | | + | | + | | - | + | + | + | + | 8 |
| (48) | | Kluit, 2022 | + | + | + | | + | | + | | - | + | + | + | + | 9 |
| (49) | | Knott, 2014 | + | + | ? | | + | | + | | ? | + | + | + | + | 8 |
| (50) | | Libeson, 2018 | + | + | ? | | ? | | ? | | - | - | + | + | + | 5 |
| (51) | | Lindahl, 2013 | + | + | + | | - | | ? | | + | + | + | + | + | 8 |
| (52) | | Lock, 2005 | - | + | + | | ? | | + | | + | - | + | + | + | 7 |
| (53) | | Lork, 2018 | + | + | + | | + | | + | | - | + | + | + | + | 9 |
| (54) | | Lysaght, 2008 | - | + | ? | | ? | | + | | + | + | + | + | + | 7 |
| (55) | | MacLennan, 2021 | + | + | ? | | + | | + | | + | + | + | + | + | 9 |
| (56) | | Madsen, 2023 | + | + | + | | + | | ? | | ? | + | ? | + | + | 7 |
| (57) | | Maillette, 2017 | + | + | + | | + | | + | | + | + | + | + | + | 10 |
| (58) | | Mansfield, 2014 | + | + | - | | + | | - | | - | + | + | + | + | 7 |
| (59) | | Martensson, 2012 | - | + | + | | + | | + | | + | - | + | + | + | 8 |
| (60) | | McKay, 2013 | + | + | - | | + | | ? | | - | + | + | + | + | 7 |
| (61) | | McRae, 2016 | + | + | + | | + | | + | | + | + | + | + | + | 10 |
| (62) | | Medin, 2006 | + | + | - | | + | | + | | + | - | + | + | + | 8 |
| (63) | | Miller, 2023 | + | + | ? | | ? | | ? | | ? | + | + | + | - | 5 |
| (64) | | Mussener, 2005 | + | + | + | | + | | + | | - | + | + | + | + | 9 |
| (65) | | Mussener, 2007 | + | + | + | | + | | + | | ? | + | + | + | + | 9 |
| (66) | | Netto, 2016 | + | + | + | | + | | + | | ? | + | + | + | + | 9 |
| (67) | | Newingston, 2019 | + | + | ? | | + | | + | | + | + | + | + | + | 9 |
| (68) | | Nilsson, 2011 | + | + | - | | + | | ? | | - | + | + | + | + | 7 |
| (69) | | Noordik, 2011 | + | + | - | | + | | + | | + | + | + | + | + | 9 |
| (70) | | Nouri, 2020 | + | + | + | | + | | ? | | + | + | ? | - | + | 7 |
| (71) | | Olischläger, 2022 | + | + | ? | | + | | + | | - | + | + | + | + | 8 |
| (72) | | Oosting, 2023 | + | + | ? | | + | | + | | - | + | + | + | + | 8 |
| (73) | | Oster, 2010 | + | + | + | | + | | + | | + | + | + | + | + | 10 |
| (74) | | Österholm, 2013 | + | + | ? | | + | | ? | | - | + | - | + | + | 6 |
| (75) | | Pahlplatz, 2021 | + | + | ? | | ? | | + | | - | + | - | + | - | 5 |
| (76) | | Pasanen, 2021 | + | + | ? | | ? | | + | | - | - | + | + | + | 6 |
| (77) | | Poulsen, 2022 | + | + | ? | | + | | + | | - | + | + | + | + | 8 |
| (78) | | Pourhabib, 2022 | + | + | ? | | ? | | + | | ? | + | ? | - | - | 4 |
| (79) | | Rubenson, 2007 | + | + | + | | + | | - | | - | - | + | + | + | 7 |
| (80) | | Ryan, 2014 | + | + | + | | + | | + | | + | + | + | + | + | 10 |
| (81) | | Sarfo, 2022 | + | + | ? | | + | | + | | + | + | + | + | + | 9 |
| (82) | | Shaw, 2003 | + | + | + | | + | | + | | - | + | + | + | + | 9 |
| (83) | | Shaw, 2009 | + | + | + | | + | | + | | + | ? | + | + | + | 9 |
| (84) | | Sjo strom, 2011 | + | + | ? | | - | | ? | | - | + | + | + | + | 6 |
| (85) | | Soeker, 2008 | + | + | + | | + | | + | | + | + | + | + | + | 10 |
| (86) | | Sturesson, 2014 | + | + | ? | | + | | + | | - | + | + | + | + | 8 |
| (87) | | Svensson, 2003 | + | + | ? | | + | | + | | - | + | + | + | + | 8 |
| (88) | | Tamminga, 2012 | + | + | + | | + | | + | | + | + | + | + | + | 10 |
| (89) | | Urquhart, 2022 | + | + | + | | + | | + | | - | + | + | + | + | 9 |
| (90) | | Van der Meer, 2011 | + | + | ? | | + | | + | | + | + | - | + | + | 8 |
| (91) | | Van Egmond, 2017 | ? | + | ? | | + | | + | | + | + | + | + | + | 8 |
| (92) | | Vooijs, 2017 | + | + | + | | + | | + | | + | + | + | + | + | 10 |
| (93) | | Wallstedt-Paulsson, 2007 | + | + | + | | - | | + | | - | + | + | + | - | 7 |
| (94) | | Watter, 2021 | + | + | + | | + | | - | | - | + | + | + | + | 8 |
| (95) | | Yarker, 2010 | + | + | + | | + | | + | | - | ? | + | + | + | 8 |
| (96) | | Zaman, 2017 | + | + | + | | ? | | + | | + | + | + | + | + | 9 |
| (97) | | Zamanzadeh, 2018 | + | + | ? | | - | | + | | - | + | + | + | + | 7 |

Supplemental material: Appendix Table 4 – Evidence profile table of the identified needs in work-focused healthcare

| # | Summarized review finding | Methodological limitations | Coherence | Adequacy | Relevance | GRADE-CERQual assessment of confidence | References |
| --- | --- | --- | --- | --- | --- | --- | --- |
| Substantive guidance | | | | | | | |
| 1.1 | **Work as a topic in healthcare delivery** - Need for work-focused support by all professionals throughout the healthcare delivery process, including the medical specialist and rehabilitation professional, to facilitate staying at work or returning to work. | Minor concerns  **Explanation:** Minor concerns regarding methodological limitations because the relationship between the researchers and participants were often unknown: a physician in the role of the researcher may have influenced the answers of patients due to participant bias. | No/Very minor concerns | No/Very minor concerns | No/Very minor concerns | High confidence  **Explanation:** Minor concerns regarding methodological limitations, No/Very minor concerns regarding coherence, No/Very minor concerns  regarding adequacy, and No/Very minor concerns regarding relevance | (4, 7-9, 11-16, 19, 21, 23, 27, 30, 32, 38, 40, 41, 45, 46, 48, 50-54, 57, 62, 63, 70, 72, 73, 75, 80, 88-90, 92, 94, 97) |
| 1.2 | **Practical and specific guidance** - Need to receive practical tips, e.g. on work modifications, and targeted and phased rehabilitation and return to work plans including realistic goals, in order to help the patient avoid exceeding their limits | No/Very minor concerns | No/Very minor concerns | No/Very minor concerns  . | Minor concerns  **Explanation:** Minor concerns regarding relevance because the studies came from a small range of geographical, high-income settings. | High confidence  **Explanation:** No/Very minor concerns regarding methodological limitations, No/Very minor concerns regarding coherence, No/Very minor concerns  regarding adequacy, and Minor concerns regarding relevance | (4, 7, 9, 10, 14, 18-20, 22, 25, 27, 29-32, 35, 38, 49-51, 55, 57, 58, 61, 62, 66-68, 70, 73, 79, 86, 88, 90, 92, 96) |
| 1.3 | **Psychological support** - Need for psychological assessment and support to help process the impact of the medical condition on impairment in living and working. | No/Very minor concerns | No/Very minor concerns | No/Very minor concerns | Minor concerns  **Explanation:** Minor concerns regarding relevance because the studies came from a small range of geographical, high-income settings. | High confidence  **Explanation:** No/Very minor concerns regarding methodological limitations, No/Very minor concerns regarding coherence, No/Very minor concerns regarding adequacy, and Minor concerns regarding relevance | (13, 15, 16, 18, 28, 38, 39, 41, 45, 47, 50, 52, 55, 58, 61, 69, 73, 76, 92, 95) |
| 1.4 | **Vocational rehabilitation** - Need for vocational rehabilitation to gain insight into and restore functional abilities and to explore suitable work arrangements. | No/Very minor concerns | Moderate concerns  **Explanation:** Moderate concerns regarding coherence due to an apparent difference in needs between unemployed and employed patients regarding vocational rehabilitation: unemployed patients focus more on exploring suitable work arrangements, while employed patients focus more on restoring own functional abilities within the old function. | No/Very minor concerns | Minor concerns  **Explanation:** Minor concerns regarding relevance because the studies came from a small range of geographical, high-income settings. | Moderate confidence  **Explanation:** No/Very minor concerns regarding methodological limitations, Moderate concerns regarding coherence, No/Very minor concerns regarding adequacy, and Minor concerns regarding relevance | (3, 15, 20, 22, 32, 33, 35, 36, 57, 73, 92-94) |
| Clear and continuous process | | | | | | | |
| 2.1 | **Early access to support** - Need for early presence and access to work-focused healthcare support, by being able to easily reach out and make timely appointments with relevant professionals. | No/Very minor concerns | Minor concerns  **Explanation:** Minor concerns regarding coherence because it was not entirely clear why patients in one study reported early invitation to occupational health as distrust, whereas the patients in the other studies perceived the need for early access to work-focused healthcare. | No/Very minor concerns | Minor concerns  **Explanation:** Minor concerns regarding relevance because the studies came from a small range of geographical, high-income settings. | Moderate confidence  **Explanation:** No/Very minor concerns regarding methodological limitations, Minor concerns regarding coherence, No/Very minor concerns regarding adequacy, and Minor concerns regarding relevance | (15, 16, 20, 28, 31, 32, 35, 38-41, 52, 57, 59, 61, 64, 66, 69, 71, 74, 86, 92, 94-96) |
| 2.2 | **Continuity in support** - Need for continuous work-focused consultations, including continuous presence of support after full return to work, and the option to fall back on someone when struggling with work participation problems. | No/Very minor concerns | Minor concerns  **Explanation:** Minor concerns regarding coherence because it was not entirely clear why patients in one study reported continuous consultations as unnecessary, whereas the patients in the other studies perceived the need for continuity in support. | No/Very minor concerns | Minor concerns  **Explanation:** Minor concerns regarding relevance because studies came from a small range of geographical, high-income settings. | Moderate confidence  **Explanation:** No/Very minor concerns regarding methodological limitations, Minor concerns regarding coherence, No/Very minor concerns  regarding adequacy, and Minor concerns regarding relevance | (6, 7, 9, 13, 14, 16, 28, 31, 33, 36-39, 41-45, 47, 48, 52, 56, 59, 61, 66, 70, 73, 76, 78, 81, 85, 86, 89-91, 93-95) |
| 2.3 | **Transparency in the process steps** - Need for transparency in the multiple process steps, for example by offering a clear overview of the role and responsibility of each professional in the process and clear feedback on how decisions affect the process. | No/Very minor concerns | No/Very minor concerns | No/Very minor concerns | Minor concerns  **Explanation:** Minor concerns regarding relevance because the studies came from a small range of geographical, high-income settings. | High confidence  **Explanation:** No/Very minor concerns regarding methodological limitations, No/Very minor concerns regarding coherence, No/Very minor concerns regarding adequacy, and Minor concerns regarding relevance | (6, 13, 19-21, 27, 30, 33, 35-37, 39, 43, 59, 61, 76, 83, 86, 94-96) |
| 2.4 | **Interdisciplinary teamwork and coordination** - Need for coherent interaction and constructive collaboration between professionals involved in work-focused healthcare, as well as towards the employer. Involvement of an independent mediator to coordinate the process is suggested. | No/Very minor concerns | No/Very minor concerns | No/Very minor concerns | No/Very minor concerns | High confidence  **Explanation:** No/Very minor concerns regarding methodological limitations, No/Very minor concerns regarding coherence, No/Very minor concerns  regarding adequacy, and No/Very minor concerns regarding relevance | (1, 3, 6, 8, 9, 11, 13-17, 19, 20, 28, 31-34, 37-40, 44, 45, 47-50, 52-56, 58, 60-62, 65, 66, 69-72, 77, 81-83, 85, 86, 89, 94, 95) |
| 2.5 | **Information about the rights and regulations** - Need for a clear overview of rights and regulations regarding the work-focused healthcare process and the patient’s obligations, in different formats at multiple time points throughout the process. | No/Very minor concerns | No/Very minor concerns | No/Very minor concerns | Serious concerns  **Explanation:** Serious concerns regarding relevance because the studies came from a small range of geographical, high-income settings; most studies described the need for information provision only during the SSA trajectory concerning the disability benefit process, and the need for information provision in different formats and at multiple time points is not specified in relation to the information provision on rights and regulations. | Low confidence  **Explanation:** No/Very minor concerns regarding methodological limitations, No/Very minor concerns regarding coherence, No/Very minor concerns regarding adequacy, and Serious concerns regarding relevance | (6, 14, 15, 22, 24, 27, 28, 34, 39, 41, 48, 49, 59, 66, 68, 70, 83, 90, 92, 94) |
| Supportive attitude and behavior | | | | | | | |
| 3.1 | **Trustful relationship** - Need for a trustful relationship with the professional, developed by being treated with respect, taken seriously, being trusted and an emphatic and in-person approach from the professional. | No/Very minor concerns | Minor concerns  **Explanation:** Minor concerns regarding coherence because the balance between the need for empathy and compassion as mentioned in multiple studies and the need for a strict attitude by the professional as mentioned in one study is not entirely clear. | No/Very minor concerns | Minor concerns  **Explanation:** Minor concerns regarding relevance because the studies came from a small range of geographical, high-income settings. | Moderate confidence  **Explanation:** No/Very minor concerns regarding methodological limitations, Minor concerns regarding coherence, No/Very minor concerns regarding adequacy, and Minor concerns regarding relevance | (1, 8, 13, 16, 20, 25, 31, 34, 39, 48, 57, 59, 63-65, 68, 73, 75, 76, 79, 84-87, 91-93, 96) |
| 3.2 | **Motivational attitude** - Need for an encouraging, positive, and proactive attitude from professionals, by sharing positive thoughts about the patient’s abilities, to motivate the patient to return to work. | No/Very minor concerns | No/Very minor concerns | No/Very minor concerns | Minor concerns  **Explanation:** Minor concerns regarding relevance because the studies came from a small range of geographical, high-income settings. | High confidence  **Explanation:** No/Very minor concerns regarding methodological limitations, No/Very minor concerns regarding coherence, No/Very minor concerns regarding adequacy, and Minor concerns regarding relevance | (1, 9, 12-15, 19, 23, 24, 30, 50, 52, 53, 59, 63-65, 67-69, 73, 76, 79, 86-88, 90-92, 96) |
| 3.3 | **Equal partnership** - Need for an equal partnership, with equal power dynamics, between the professional and patient in making decisions regarding vocational reintegration, by listening and valuing the patient’s choices. | No/Very minor concerns | Minor concerns  **Explanation:** Minor concerns regarding coherence because the balance between the need for decision-making and relieve from the responsibility in decision-making by the patient was not entirely clear. | No/Very minor concerns | Minor concerns  **Explanation:** Minor concerns regarding relevance because the studies came from a small range of geographical, high-income settings. | Moderate confidence  **Explanation:** No/Very minor concerns regarding methodological limitations, Minor concerns regarding coherence, No/Very minor concerns  regarding adequacy, and Minor concerns regarding relevance | (5, 6, 15-17, 21, 30, 34, 38, 39, 42, 50, 55, 57, 59, 61, 62, 64-67, 71, 76, 81, 86, 87, 93) |
| 3.4 | **Patient advocacy** - Need for the professional to act in the patient’s interests instead of in the interests of other parties, such as the employer. | No/Very minor concerns | No/Very minor concerns | Moderate concerns  **Explanation:** Moderate concerns regarding adequacy because of few studies and very thin data. | No/Very minor concerns | Moderate confidence  **Explanation:** Minor concerns regarding methodological limitations, No/Very minor concerns regarding coherence, Moderate concerns regarding adequacy, and No/Very minor concerns regarding relevance | (5, 15, 20, 25, 40, 59, 64, 89, 92, 96) |
| Tailored approach | | | | | | | |
| 4.1 | **Flexibility in work-focused healthcare** - Need for flexibility in the work-focused healthcare provision, and flexibility in the application of the rules in the context of the patient’s needs, in order to receive more tailored support. | No/Very minor concerns | No/Very minor concerns | No/Very minor concerns | Minor concerns  **Explanation:** Minor concerns regarding relevance because the studies came from a small range of geographical, high-income settings. | High confidence  **Explanation:** No/Very minor concerns regarding methodological limitations, No/Very minor concerns regarding coherence, Minor concerns regarding adequacy, and Minor concerns regarding relevance | (1, 5-7, 9, 12, 15-17, 20, 21, 25, 26, 31, 34-36, 42-44, 46, 48, 49, 51-53, 57, 59, 61, 62, 65, 66, 68, 71, 73, 76, 78, 86, 87, 89, 91-94) |
| 4.2 | **Attention for the personal situation** - Need for attention for the personal situation, including understanding of work capabilities and knowledge of the specific medical situation, on the part of the professional. | No/Very minor concerns | No/Very minor concerns | No/Very minor concerns | Minor concerns  **Explanation:** Minor concerns regarding relevance because most studies came from a small range of geographical, high-income settings. | High confidence  **Explanation:** No/Very minor concerns regarding methodological limitations, No/Very minor concerns regarding coherence, No/Very minor concerns regarding  adequacy, and Minor concerns regarding relevance | (1, 5, 9, 10, 12, 13, 15-17, 19, 22, 24, 25, 28, 30, 31, 33, 38-40, 44, 47, 50, 53, 58, 59, 61, 66-73, 76, 79, 81, 84-86, 88, 89, 92, 94, 96) |
| 4.3 | **Inclusion of patient-focused goals** - Need for professionals to include patient-focused goals, meeting the patient’s own goals and motivation. | No/Very minor concerns | No/Very minor concerns | Minor concerns  **Explanation:** Minor concerns regarding adequacy because given the large amount of included studies, this specific need is underrepresented in the data. | Minor concerns  **Explanation:** Minor concerns regarding relevance because the studies came from a small range of geographical, high-income settings. | Moderate confidence  **Explanation:** No/Very minor concerns regarding methodological limitations, No/Very minor concerns regarding coherence, Minor concerns regarding adequacy, and Minor concerns regarding relevance | (7, 13, 15-19, 30, 58, 59, 62, 67, 72, 76, 80, 84, 90) |
| 4.4 | **Disease-specific information in relation to work** - Need for information provision on the expected disease-specific consequences on work, such as expected return to work timelines and impact on work-capacity due to the diagnosis. | No/Very minor concerns | No/Very minor concerns | No/Very minor concerns | No/Very minor concerns | High confidence  **Explanation:** No/Very minor concerns regarding methodological limitations, No/Very minor concerns regarding coherence, No/Very minor concerns regarding adequacy, and No/Very minor concerns regarding relevance | (2, 7, 16, 18, 24, 27-29, 31, 35, 40, 47, 48, 54, 56, 59, 62, 68, 70-75, 79, 83, 90-92, 94, 95, 97) |

**REFERENCES**

1. Aamland A, Werner EL, Malterud K. Sickness absence, marginality, and medically unexplained physical symptoms: a focus-group study of patients’ experiences. Scandinavian journal of primary health care. 2013;31(2):95-100.

2. Abma FI, Bültmann U, Varekamp I, van der Klink JJ. Workers with health problems: three perspectives on functioning at work. Disability and Rehabilitation. 2013;35(1):20-6.

3. Aguiar-Fernández F, Rodríguez-Castro Y, Botija M, Martínez-Román R. Experiences of female breast cancer survivors concerning their return to work in Spain. Behavioral Sciences. 2021;11(10):135.

4. Amir Z, Neary D, Luker K. Cancer survivors’ views of work 3 years post diagnosis: a UK perspective. European Journal of Oncology Nursing. 2008;12(3):190-7.

5. Andersson C, Mårtensson L. Womenʼs experiences of being in the sick leave process. Scandinavian journal of occupational therapy. 2021;28(6):488-97.

6. Andersson C, Jakobsson A, Priebe G, Elf M, Fornazar R, Hensing G. Capability to make well-founded decisions: an interview study of people with experience of sickness absence who have common mental disorders. BMC Public Health. 2022;22(1):1189.

7. Audhoe SS, Nieuwenhuijsen K, Hoving JL, Sluiter JK, Frings-Dresen MH. Perspectives of unemployed workers with mental health problems: barriers to and solutions for return to work. Disability and rehabilitation. 2018;40(1):28-34.

8. Bae KR, Cho J. Changes after cancer diagnosis and return to work: experience of Korean cancer patients. BMC cancer. 2021;21(1):1-11.

9. Bardgett M, Lally J, Malviya A, Deehan D. Return to work after knee replacement: a qualitative study of patient experiences. BMJ open. 2016;6(2):e007912.

10. Beaulieu K. A new conceptual framework to facilitate return to paid work following a brain injury. 2016.

11. Beerda DC, Zegers AD, van Andel ES, Becker-Commissaris A, van der Vorst MJ, Tange D, et al. Experiences and perspectives of patients with advanced cancer regarding work resumption and work retention: a qualitative interview study. Supportive Care in Cancer. 2022;30(12):9713-21.

12. Bennink C, van Der Klift M, Scheurer H, Sonneveld P, Duijts SF. Perspectives on returning to work of multiple myeloma patients: A qualitative interview study. European Journal of Cancer Care. 2021;30(6):e13481.

13. Berger I, Beck L, Jones J, MacEachen E, Kirsh B. Exploring the Needs of Cancer Survivors When Returning to or Staying in the Workforce. Journal of occupational rehabilitation. 2020;30(3):480-95.

14. Blokzijl F, Onrust M, Dieperink W, Keus F, van der Horst IC, Paans W, et al. Barriers that obstruct return to work after coronary bypass surgery: a qualitative study. Journal of occupational rehabilitation. 2021;31(2):316-22.

15. Bosma A, Boot C, Schaafsma F, Anema J. Facilitators, barriers and support needs for staying at work with a chronic condition: a focus group study. BMC public health. 2020;20(1):1-11.

16. Brakenridge CL, Leow CKL, Kendall M, Turner B, Valiant D, Quinn R, et al. Exploring the lived return-to-work experience of individuals with acquired brain injury: use of vocational services and environmental, personal and injury-related influences. Disability and rehabilitation. 2021:1-11.

17. Bratun U, Švajger A, Domajnko B, Kavčič M, Asaba E. Return to work among workers recovering from severe COVID-19 in Slovenia: a focus group study. Disability and Rehabilitation. 2022:1-10.

18. Bridger K, Kellezi B, Kendrick D, Radford K, Timmons S, Rennoldson M, et al. Patient perspectives on key outcomes for vocational rehabilitation interventions following traumatic injury. International journal of environmental research and public health. 2021;18(4):2035.

19. Coole C, Watson PJ, Drummond A. Staying at work with back pain: patients' experiences of work-related help received from GPs and other clinicians. A qualitative study. BMC Musculoskeletal Disorders. 2010;11(1):1-7.

20. Coole C, Watson PJ, Drummond A. Low back pain patients' experiences of work modifications; a qualitative study. BMC musculoskeletal disorders. 2010;11(1):1-10.

21. Corbière M, Charette-Dussault É, Larivière N. Recognition During the Return-to-Work Process in Workers with Common Mental Disorders. Journal of Occupational Rehabilitation. 2022:1-20.

22. Decuman S, Smith V, Grypdonck M, De Keyser F, Verhaeghe S. Factors influencing the occupational trajectory of patients with systemic sclerosis: a qualitative study. Clin Exp Rheumatol. 2015;33(Suppl 91):S26-30.

23. Dewa CS, Trojanowski L, Tamminga SJ, Ringash J, McQuestion M, Hoch JS. Work-related experiences of head and neck cancer survivors: an exploratory and descriptive qualitative study. Disability and rehabilitation. 2018;40(11):1252-8.

24. Donker-Cools BH, Schouten MJ, Wind H, Frings-Dresen MH. Return to work following acquired brain injury: the views of patients and employers. Disability and rehabilitation. 2018;40(2):185-91.

25. Dorland H, Abma F, Roelen C, Smink J, Ranchor A, Bültmann U. Factors influencing work functioning after cancer diagnosis: a focus group study with cancer survivors and occupational health professionals. Supportive Care in Cancer. 2016;24(1):261-6.

26. Duijts SF, van Egmond MP, Gits M, van der Beek AJ, Bleiker EM. Cancer survivors’ perspectives and experiences regarding behavioral determinants of return to work and continuation of work. Disability and rehabilitation. 2017;39(21):2164-72.

27. Frazier LM, Miller VA, Miller BE, Horbelt DV, Delmore JE, Ahlers-Schmidt CR. Cancer-related tasks involving employment: opportunities for clinical assistance. The journal of supportive oncology. 2009;7(6):229.

28. Gard G, Pessah-Rasmussen H, Brogårdh C, Nilsson Å, Lindgren I. Need for structured healthcare organization and support for return to work after stroke in Sweden: Experiences of stroke survivors. Journal of rehabilitation medicine. 2019;51(10):741-8.

29. Gilworth G, Eyres S, Carey A, Bhakta B, Tennant A. Working with a brain injury: personal experiences of returning to work following a mild or moderate brain injury. Journal of Rehabilitation Medicine. 2008;40(5):334-9.

30. Gilworth G, Phil M, Cert A, Sansam K, Kent R. Personal experiences of returning to work following stroke: an exploratory study. Work. 2009;34(1):95-103.

31. Graff HJ, Deleu NW, Christiansen P, Rytter HM. Facilitators of and barriers to return to work after mild traumatic brain injury: A thematic analysis. Neuropsychological rehabilitation. 2021;31(9):1349-73.

32. Hartke RJ, Trierweiler R, Bode R. Critical factors related to return to work after stroke: a qualitative study. Topics in stroke rehabilitation. 2011;18(4):341-51.

33. Hellman T, Bergström A, Eriksson G, Hansen Falkdal A, Johansson U. Return to work after stroke: Important aspects shared and contrasted by five stakeholder groups. Work. 2016;55(4):901-11.

34. Henry AD, Lucca AM. Facilitators and barriers to employment: The perspectives of people with psychiatric disabilities and employment service providers. Work. 2004;22(3):169-82.

35. Hjärtström C, Norberg AL, Johansson G, Bodin T. To work despite chronic health conditions: a qualitative study of workers at the Swedish Public Employment Service. BMJ open. 2018;8(4):e019747.

36. Holmlund L, Guidetti S, Eriksson G, Asaba E. Return to work in the context of everyday life 7–11 years after spinal cord injury–a follow-up study. Disability and rehabilitation. 2018;40(24):2875-83.

37. Holmlund L, Hellman T, Engblom M, Kwak L, Sandman L, Törnkvist L, et al. Coordination of return-to-work for employees on sick leave due to common mental disorders: facilitators and barriers. Disability and Rehabilitation. 2020:1-9.

38. Hooson JM, Coetzer R, Stew G, Moore A. Patients' experience of return to work rehabilitation following traumatic brain injury: A phenomenological study. Neuropsychological rehabilitation. 2013;23(1):19-44.

39. Hubertsson J, Petersson IF, Arvidsson B, Thorstensson CA. Sickness absence in musculoskeletal disorders-patients' experiences of interactions with the social insurance agency and health care. A qualitative study. BMC Public Health. 2011;11(1):1-9.

40. Jain A, Aggarwal A, Adams J, Jordan RE, Sadhra S, Dubey S, et al. Work productivity loss among rheumatoid arthritis patients in India: a qualitative study. Rheumatology advances in practice. 2019;3(2):rkz046.

41. Jansson I, Björklund A. The experience of returning to work. Work. 2007;28(2):121-34.

42. Jarman V, Hancock N, Scanlan JN. Maintaining my employment: Learning from people living and working with mental illness. British Journal of Occupational Therapy. 2016;79(11):660-8.

43. Joosen MC, Lugtenberg M, Arends I, van Gestel HJ, Schaapveld B, Terluin B, et al. Barriers and facilitators for return to work from the perspective of workers with common mental disorders with short, medium and long-term sickness absence: a longitudinal qualitative study. Journal of Occupational Rehabilitation. 2021:1-12.

44. Juurlink TT, Vukadin M, Stringer B, Westerman MJ, Lamers F, Anema JR, et al. Barriers and facilitators to employment in borderline personality disorder: A qualitative study among patients, mental health practitioners and insurance physicians. PloS one. 2019;14(7):e0220233.

45. Karcz K, Schiffmann B, Schwegler U, Staubli S, Finger ME. Facilitators and barriers to sustainable employment after spinal cord injury or acquired brain injury: the person's perspective. Frontiers in rehabilitation sciences. 2022;3:872782.

46. Kennedy F, Haslam C, Munir F, Pryce J. Returning to work following cancer: a qualitative exploratory study into the experience of returning to work following cancer. European journal of cancer care. 2007;16(1):17-25.

47. Klaver KM, Duijts SF, Engelhardt EG, Geusgens CA, Aarts MJ, Ponds RW, et al. Cancer-related cognitive problems at work: experiences of survivors and professionals. Journal of Cancer Survivorship. 2020;14(2):168-78.

48. Kluit L, de Wind A, Oosting IJ, van Velzen JM, Beumer A, Sluman MA, et al. Current practices, needs, and expectations of discussing work with a medical specialist from a patient’s perspective: a qualitative study. Disability and Rehabilitation. 2022:1-14.

49. Knott V, Zrim S, Shanahan EM, Anastassiadis P, Lawn S, Kichenadasse G, et al. Returning to work following curative chemotherapy: a qualitative study of return to work barriers and preferences for intervention. Supportive Care in Cancer. 2014;22(12):3263-73.

50. Libeson L, Downing M, Ross P, Ponsford J. The experience of return to work in individuals with traumatic brain injury (TBI): A qualitative study. Neuropsychological rehabilitation. 2020;30(3):412-29.

51. Lindahl M, Hvalsoe B, Poulsen JR, Langberg H. Quality in rehabilitation after a working age person has sustained a fracture: Partnership contributes to continuity. Work. 2013;44(2):177-89.

52. Lock S, Jordan* L, Bryan K, Maxim J. Work after stroke: focusing on barriers and enablers. Disability & society. 2005;20(1):33-47.

53. Lork K, Holmgren K. The experience of return to work self-efficacy among people on sick leave. Work. 2018;59(4):479-90.

54. Lysaght RM, Larmour-Trode S. An exploration of social support as a factor in the return-to-work process. Work. 2008;30(3):255-66.

55. MacLennan SJ, Cox T, Murdoch S, Eatough V. An interpretative phenomenological analysis of the meaning of work to women living with breast cancer. Chronic Illness. 2021:1742395320987883.

56. Madsen CMT, Christensen JR, Bremander A, Primdahl J. Perceived challenges at work and need for professional support among people with inflammatory arthritis-a qualitative interview study. Scandinavian Journal of Occupational Therapy. 2021:1-10.

57. Maillette P, Coutu M-F, Gaudreault N. Workers’ perspectives on return to work after total knee arthroplasty. Annals of physical and rehabilitation medicine. 2017;60(5):299-305.

58. Mansfield E, Stergiou-Kita M, Kirsh B, Colantonio A. After the storm: the social relations of return to work following electrical injury. Qualitative health research. 2014;24(9):1183-97.

59. Mårtensson L, Hensing G. Experiences of factors contributing to women's ability to make informed decisions about the process of rehabilitation and return to work: a focus group study. Work. 2012;43(2):237-48.

60. McKay G, Knott V, Delfabbro P. Return to work and cancer: the Australian experience. Journal of occupational rehabilitation. 2013;23(1):93-105.

61. McRae P, Hallab L, Simpson G. Navigating employment pathways and supports following brain injury in Australia: Client perspectives. The Australian Journal of Rehabilitation Counselling. 2016;22(2):76-92.

62. Medin J, Barajas J, Ekberg K. Stroke patients' experiences of return to work. Disability and rehabilitation. 2006;28(17):1051-60.

63. Miller A, Wilson E, Diver C. Returning to work: a qualitative study of the experiences of head and neck cancer survivors. The Journal of Laryngology & Otology. 2023;137(6):691-6.

64. Mussener U, Svensson T, Soderberg E, Alexanderson K. Encouraging encounters: sick-listed persons' experiences of interactions with rehabilitation professionals. Social work in health care. 2007;46(2):71-87.

65. Müssener U, Ståhl C, Söderberg E. Does the quality of encounters affect return to work? Lay people describe their experiences of meeting various professionals during their rehabilitation process. Work. 2015;52(2):447-55.

66. Netto JA, Yeung P, Cocks E, McNamara B. Facilitators and barriers to employment for people with mental illness: A qualitative study. Journal of Vocational Rehabilitation. 2016;44(1):61-72.

67. Newington L, Brooks C, Warwick D, Adams J, Walker-Bone K. Return to work after carpal tunnel release surgery: a qualitative interview study. BMC musculoskeletal disorders. 2019;20(1):1-11.

68. Nilsson M, Olsson M, Wennman-Larsen A, Petersson L-M, Alexanderson K. Return to work after breast cancer: women’s experiences of encounters with different stakeholders. European Journal of Oncology Nursing. 2011;15(3):267-74.

69. Noordik E, Nieuwenhuijsen K, Varekamp I, van der Klink JJ, J. van Dijk F. Exploring the return-to-work process for workers partially returned to work and partially on long-term sick leave due to common mental disorders: a qualitative study. Disability and rehabilitation. 2011;33(17-18):1625-35.

70. Nouri F, Coole C, Baker P, Drummond A. Return to work advice after Total Hip and Knee Replacement. Occupational Medicine. 2020;70(2):113-8.

71. Olischläger DL, den Boer LXY, de Heus E, Brom L, Dona DJ, Klümpen H-J, et al. Rare cancer and return to work: experiences and needs of patients and (health care) professionals. Disability and Rehabilitation. 2022:1-12.

72. Oosting IJ, Kluit L, Schaafsma FG, Beumer A, van Bennekom CA, de Boer AG, et al. Patients' Experiences, Needs, and Expectations of Cooperation Between Medical Specialists and Occupational Health Physicians: A Qualitative Study. Journal of Occupational and Environmental Medicine. 2023;65(6):e395.

73. Öster C, Kildal M, Ekselius L. Return to work after burn injury: burn-injured individuals' perception of barriers and facilitators. Journal of Burn Care & Research. 2010;31(4):540-50.

74. Österholm JH, Björk M, Håkansson C. Factors of importance for maintaining work as perceived by men with arthritis. Work. 2013;45(4):439-48.

75. Pahlplatz T, Schafroth M, Krijger C, Hylkema T, van Dijk C, Frings-Dresen M, et al. Beneficial and limiting factors in return to work after primary total knee replacement: Patients’ perspective. Work. 2021;69(3):895-902.

76. Pasanen J. The nature of positive encounters between disabled workers and insurers in the return to work process. Work. 2021;70(1):287-300.

77. Poulsen AG, Rolving N, Hubeishy MH, Ørtenblad L. Navigating between stakeholders in return-to-work processes: A qualitative study exploring experiences of workers on sick leave due to back pain. Work. 2023(Preprint):1-11.

78. Pourhabib A, Sabzi Z, Yazdi K, Fotokian Z. Facilitators and barriers to return to work in patients after heart surgery. Journal of Education and Health Promotion. 2022;11.

79. Rubenson C, Svensson E, Linddahl I, Björklund A. Experiences of returning to work after acquired brain injury. Scandinavian journal of occupational therapy. 2007;14(4):205-14.

80. Ryan CG, Lauchlan D, Rooney L, Hollins Martins C, Gray H. Returning to work after long term sickness absence due to low back pain–the struggle within: A qualitative study of the patient's experience. Work. 2014;49(3):433-44.

81. Sarfo M-C, van Asselt KM, Frings-Dresen MH, de Jong F, van Dijk N, de Boer AG. Views of breast cancer survivors on work participation guidance by general practitioners: a qualitative study. BMC Primary Care. 2022;23(1):152.

82. Shaw WS, Robertson MM, Pransky G, McLellan RK. Employee perspectives on the role of supervisors to prevent workplace disability after injuries. Journal of Occupational Rehabilitation. 2003;13(3):129-42.

83. Shaw L, Bondy K, Dodman J. Client insights on knowledge use and access in return to work. Canadian Journal of Occupational Therapy. 2009;76(5):359-67.

84. Sjöström R, Melin-Johansson C, Asplund R, Alricsson M. Barriers to and possibilities of returning to work after a multidisciplinary rehabilitation programme. A qualitative interview study. Work. 2011;39(3):243-50.

85. Soeker MS, Wegner L, Pretorius B. I'm going back to work: Back injured clients' perceptions and experiences of their worker roles. Work. 2008;30(2):161-70.

86. Sturesson M, Edlund C, Falkdal AH, Bernspång B. Healthcare encounters and return to work: a qualitative study on sick-listed patients’ experiences. Primary health care research & development. 2014;15(4):464-75.

87. Svensson T, Karlsson A, Alexanderson K, Nordqvist C. Shame-inducing encounters. Negative emotional aspects of sickness-absentees' interactions with rehabilitation professionals. Journal of occupational rehabilitation. 2003;13(3):183-95.

88. Tamminga SJ, De Boer AG, Verbeek JH, Frings-Dresen MH. Breast cancer survivors' views of factors that influence the return-to-work process-a qualitative study. Scandinavian journal of work, environment & health. 2012:144-54.

89. Urquhart R, Scruton S, Kendell C. Understanding cancer survivors’ needs and experiences returning to work post-treatment: A longitudinal qualitative study. Current Oncology. 2022;29(5):3013-25.

90. Van der Meer M, Hoving JL, Vermeulen MI, Herenius MM, Tak PP, Sluiter JK, et al. Experiences and needs for work participation in employees with rheumatoid arthritis treated with anti-tumour necrosis factor therapy. Disability and rehabilitation. 2011;33(25-26):2587-95.

91. Van Egmond M, Duijts S, Loyen A, Vermeulen S, Van der Beek A, Anema J. Barriers and facilitators for return to work in cancer survivors with job loss experience: a focus group study. European journal of cancer care. 2017;26(5):e12420.

92. Vooijs M, Leensen MC, Hoving JL, Wind H, Frings-Dresen MH. Perspectives of people with a chronic disease on participating in work: a focus group study. Journal of occupational rehabilitation. 2017;27(4):593-600.

93. Wallstedt‐Paulsson E, Erlandsson LK, Eklund M. Client experiences in work rehabilitation in Sweden: a one‐year follow‐up study. Occupational therapy international. 2007;14(1):28-41.

94. Watter K, Kennedy A, McLennan V, Vogler J, Jeffery S, Murray A, et al. Consumer perspectives of vocational rehabilitation and return to work following acquired brain injury. Brain Impairment. 2021:1-21.

95. Yarker J, Munir F, Bains M, Kalawsky K, Haslam C. The role of communication and support in return to work following cancer‐related absence. Psycho‐Oncology. 2010;19(10):1078-85.

96. Zaman A, Bruinvels D, de Boer A, Frings‐Dresen M. Supporting cancer patients with work‐related problems through an oncological occupational physician: a feasibility study. European journal of cancer care. 2017;26(5):e12378.

97. Zamanzadeh V, Valizadeh L, Rahmani A, Zirak M, Desiron H. Cancer survivors' experiences of return to work: A qualitative study. Psycho‐oncology. 2018;27(10):2398-404.
